# Predicting tumour and organ dose in α-radiopharmaceutical therapy: a ^89^Zr-based dosimetric approach for ^225^Ac

**DOI:** 10.1007/s00259-026-08076-x

**Published:** 2026-07-15

**Authors:** Robin I.J. Merkx, Simone C. Kleinendorst, Mark Rijpkema, Gerben M. Franssen, Alfred Morgenstern, Frank Bruchertseifer, Michael P. Wheatcroft, Egbert Oosterwijk, Peter F.A. Mulders, Mark Konijnenberg, Sandra Heskamp

**Affiliations:** 1 Department of Medical Imaging: Nuclear Medicine, Radboudumc, Geert Grooteplein Zuid 10, Nijmegen, 6525 GA The Netherlands; 2 Department of Urology, Radboudumc, Nijmegen, The Netherlands; 3 Joint Research Centre, European Commission, Karlsruhe, Germany; 4 Telix Pharmaceuticals Limited, Melbourne, Australia; 5 ErasmusMC, Rotterdam, The Netherlands

**Keywords:** Radiopharmaceutical therapy, RCC, Actinium-225, Zr89, Dosimetry

## Abstract

**Abstract:**

The aim of this study was to assess whether zirconium-89 (^89^Zr)-labelled antibodies can act as a surrogate for actinium-225 (^225^Ac)-labelled antibodies to predict tumour- and organ dose. To this end, we performed ex vivo biodistribution and dosimetry studies with two anti-CAIX antibodies (hG250 and FcRn(-)hG250), radiolabelled with ^89^Zr and ^225^Ac, in a renal cell carcinoma mouse model.

**Methods:**

BALB/c nude mice bearing subcutaneous SK-RC-52 tumours were injected intravenously with 25–50 kBq ^225^Ac-labelled or 1 MBq ^89^Zr-labelled anti-CAIX antibody, at equal protein dose. Organ and tumour absorbed doses were calculated based on the uptake data at 4, 24, 72, and 168 h for FcRn(-)hG250 and 24, 72, and 168 h for hG250. The predictive value of ^89^Zr for ^225^Ac was evaluated in both groups, by comparing the predicted ^225^Ac absorbed dose (based on ^89^Zr-antibody) and the measured ^225^Ac absorbed dose.

**Results:**

The biodistribution profile of the theranostic pair ^89^Zr/^225^Ac was comparable for FcRn(-)hG250. For hG250, significant discrepancies between tumour, spleen and liver uptake were observed. The predicted ^225^Ac tumour dose was overestimated by 3.9 ± 2.0 Gy (6 ± 50.6%) and underestimated by 1413.7 ± 629.1 Gy (69 ± 44.5%) in the FcRn(-)hG250 and hG250 groups, respectively. The deviation of the predicted versus measured ^225^Ac-dose in off-target organs was most notable in liver and spleen for both antibodies.

**Conclusion:**

This study highlights variability in the predictive accuracy of the ^89^Zr/^225^Ac theranostic pair for tumour and organ dosimetry in a ccRCC mouse model, depending on the antibody used. FcRn(-)hG250 enabled accurate tumour dose estimation, whereas hG250 showed mismatched uptake limiting prediction, possibly due to inherent differences in the properties and biological effects of both ^225^Ac and ^89^Zr. These findings underscore the need to further investigate how ^89^Zr-PET can be used for ^225^Ac-TRT dose prediction.

**Supplementary Information:**

The online version contains supplementary material available at https://doi.org/10.1007/s00259-026-08076-x.

## Background

Targeted radionuclide therapy (TRT) is a rapidly advancing therapeutic modality based on the delivery of radioisotopes to tumour-specific targets [1]. The specificity of this treatment in combination with the potential to visualize and monitor the radiopharmaceuticals’ distribution offers a unique opportunity for patient-tailored treatment for patient selection and personalized dosing. The interest in TRT significantly increased because of the development of novel tumour-specific targeting agents and increased availability of α- and β-emitting radioisotopes (e.g. ^177^Lu, ^161^Tb, ^153^Sm, ^212^Pb, ^225^Ac, ^227^Th) [2–6].

The favourable characteristics of α-particles, including their limited range, high linear energy transfer, and their ability to circumvent tumour-specific biological resistance mechanisms, renders targeted α therapy (TAT) a promising therapeutic modality [7]. However, it has yet to establish a firm foothold in the clinical repertoire of anticancer treatments [1, 8, 9]. One of the challenges in the clinical implementation of TAT involves the distribution of the mother (e.g. ^225^Ac and ^227^Th) and daughter (e.g. ^213^Bi, ^223^Ra) α-emitting radionuclides. Imaging and dosimetry of TAT will provide key insights to develop effective and safe treatment schedules for patients [10]. However, imaging-based dosimetry-driven treatment planning of TAT is challenging, mostly due to the low activity of α-emitters needed for treatment as well as the low yield of γ-emitting daughter radionuclides. Therefore, readily achievable quantitative three-dimensional (3D) imaging is not yet available [11]. Fortunately, innovations in single-photon emission computed tomography (SPECT) camera configuration have demonstrated the potential to enable quantitative imaging of ^223^Ra, ^225^Ac, and ^227^Th in phantom-based models [12–14]. Additionally, ongoing research is focusing on developing new reconstruction protocols aimed at enhancing the clinical applicability of TAT SPECT imaging [15–17].

An alternative solution is the adoption of surrogate imaging using PET radiopharmaceuticals (e.g. ^89^Zr-labelled radiopharmaceuticals) to address the lack of straightforward quantitative SPECT-based 3D imaging in TAT [18]. An advantage of this approach is the possibility for pre-treatment imaging, rather than mere intra-therapeutic assessment. This may enable patient selection and individualized treatment planning. However, the chosen surrogate radionuclide can significantly impact the pharmacokinetics and pharmacodynamics of radiopharmaceuticals, including tumour uptake, normal-organ distribution, and absorbed dose profiles, as previously demonstrated for ^177^Lu- versus ^225^Ac-labelled hG250 [19]. This aspect becomes particularly important for alpha-emitting radionuclides such as ^225^Ac, where recoil from multiple α-decays results in daughter radionuclides released from the radiopharmaceutical which potentially redistribute to healthy non-target tissues [20]. Such intricacies warrant careful consideration during PET-based treatment planning.

A target that has been extensively investigated in the setting of both PET-imaging as well as TRT is carbonic anhydrase IX (CAIX), a tumour-associated antigen overexpressed in clear cell renal cell carcinoma (ccRCC) [21]. In a recent phase III study, the accuracy and safety of [^89^Zr]Zr-DFO-cG250 (girentuximab) PET/CT have been shown in patients with ccRCC [22–24]. However, clinical implementation of TRT using ^177^Lu-cG250 in ccRCC has been hampered due to bone marrow toxicity, demanding the search for a more effective, safe, and patient-tailored approach, such as dosimetry-based TAT [25].

Hence, we aimed to assess the feasibility of employing positron-emitting ^89^Zr as a surrogate imaging radionuclide for ^225^Ac for two antibody constructs targeting CAIX in a mouse model for ccRCC. These constructs encompass a humanized monoclonal antibody, hG250, and a novel version of this antibody, FcRn(-)hG250. The latter has been modified to decrease neonatal Fc-receptor (FcRn)-IgG interaction, allowing for faster clearance and potentially decreasing toxicity. In this study we investigated the potential of ^89^Zr-labelled antibodies (hG250 and FcRn(-)hG250) to predict the tumour and healthy tissue absorbed doses of their ^225^Ac-labelled counterparts.

## Material & methods

### Antibodies & cell line

Humanized G250 (hG250) is an IgG1 monoclonal antibody that is modified without adapting the binding pocket of the original chimeric G250. It is harvested from hamster ovary cells and is directed against the CAIX antigen. HG250 was received from Telix Pharmaceuticals Ltd. (Melbourne, Victoria, Australia). Fc-silent humanized G250 (FcRn(-)hG250) was obtained by reducing the affinity of the antibody for the neonatal Fc receptor (FcRn) of the humanized G250 through amino acid substitutions H310A and 435Ω and was also received from Telix Pharmaceuticals Ltd.

The CAIX-expressing RCC cell line SK-RC-52 (RRID: CVCL_6198) was derived from a mediastinal metastasis of a primary RCC. SK-RC-52 cells were cultured in RPMI1640 medium, supplemented with 10% fetal calf serum (FCS) at 37 °C in a humidified atmosphere with 5% CO2. Prior to in vitro or in vivo experiments, cells were washed with saline, trypsinized, and washed with RPMI-1640 10% FCS.

### Conjugation, labelling & quality control

FcRn(-)hG250 and hG250 were conjugated with DFO or DOTA chelators as described previously [22, 26]. Radiolabeling with ^89^Zr or ^225^Ac was performed under optimized conditions for each antibody, and radiochemical purity (> 95%) was confirmed by ITLC. Detailed conjugation and labelling procedures are provided in the Supplemental Methods. Specific activities were 0.045 and 0.073 MBq/µg, 0.55 and 3.1 kBq/µg for ^89^Zr-FcRn(-)hG250, -hG250 and ^225^Ac-FcRn(-)hG250, -hG250, respectively.

### Animal experiments

Animal experiments were conducted in accordance with the principles laid out by the Dutch Act on Animal Experimentation and approved by the institutional Animal Welfare Committee of the Radboud University Nijmegen. All experiments were performed in female BALB/cAnNRj-Foxn1^nu/nu^ mice (Janvier, le Genest-Saint-Isle, France). Mice were accustomed to laboratory conditions for one week and housed in individualized ventilated cages with ad libitum access to animal chow and water. All mice were engrafted subcutaneously with 2.5 × 10^6^ SK-RC-52 cells in 0.2 mL of RPMI-1640 in the right flank. Experiments started three weeks after tumour cell inoculation. Mice were randomly assigned to groups by block randomization based on tumour size. The biotechnicians were blinded for group allocation.

### Biodistribution study

Forty tumour-bearing mice were randomized into 8 groups. Mice were injected intravenously with a diagnostic activity of 2.3 MBq ^89^Zr- or a therapeutic activity of 25 kBq ^225^Ac-labelled FcRn(-)hG250 (50 µg). Mice were sacrificed at 4 h, 1, 3, or 7 days post injection (p.i.) for ex vivo biodistribution. The tumour and healthy tissues were harvested, weighed and counted in a γ-counter (1480 Wizard 3; LKB/Wallace, Perkin Elmer, Boston, MA), using ^221^Fr as a surrogate for ^225^Ac after > 1 h after dissection (at ^225^Ac-^221^Fr equilibrium) [27]. To express the activity in samples as percent injected activity per gram tissue (%IA/g), three aliquots containing 1% of the injected dose were counted simultaneously. Values are reported as mean ± standard deviation (SD).

To estimate the in vivo (re)biodistribution of ^213^Bi (440 keV, counting window 380–520 keV), kidneys were transferred to the γ-counter immediately after dissection and measured continuously. Next, the total ^213^Bi activity in kidneys at time of sacrifice (t = 0) was determined by using the function as described in Supplemental Material.

The biodistribution of ^225^Ac-hG250 and ^89^Zr-hG250 was performed as described previously [19]. In short, thirty tumour bearing mice were randomized into six groups through block randomization. Mice were injected intravenously with 1.2 MBq ^89^Zr-hG250 or 50 kBq ^225^Ac-hG250 (30 µg). Mice were sacrificed at 1, 3, or 7 days p.i. and the activity in samples were obtained as described above.^225^Ac-hG250 biodistribution data has been published previously [19].

Biodistribution data of ^89^Zr-FcRn(-)hG250, ^225^Ac-FcRn(-)hG250, ^89^Zr-hG250 and ^225^Ac-hG250 (data presented in Supplemental Tables 3–4) was used as input for organ and tumour dosimetry as described below.

### Organ and tumour dosimetry

#### ^*225*^*Ac-dosimetry*

The time activity curve (TAC) for all dissected organs was obtained by plotting the corresponding relative uptake at all timepoints. The TACs of all radioimmunoconjugates were fitted both to a mono- or bi-exponential function and the best fit was obtained by applying the Akaike Information Criteria. Subsequently, the time-integrated activity coefficient (TIAC) was obtained by integration of the (bi)exponential fits folded with the Ac-225 decay function and whenever the R^2^ of the fits were below 0.7, the TIAC was obtained by using the trapezoid rule. The obtained TIACs for all organs were subsequently entered as an input to OLINDA/EXM 2.1 for dose calculations. With the exception of spleen, organ masses were not adapted to the individual mouse weights, but the 25 g mice model was used. For tumour and spleen mass, the corresponding mean weight per group were used (Supplemental Tables 3–4). The tumour absorbed doses were determined using a spherical model incorporated in OLINDA/EXM 2.1. Organ- and tumour specific dose were calculated as Gy/MBq by using the MIRD formalism, taking cross-doses from surrounding tissues into account [28]. Propagation of errors in the dosimetry calculation scheme was performed according to the EANM guidance document [29]. For the dose conversion in mice that received ^225^Ac, the sum of the dose conversion factors of all daughter nuclides was used. Absolute and percent difference between predicted and measured doses (for 25 or 50 kBq ^225^Ac) were determined, and their SD was determined by the square root of the sum of the %SD of predicted and measured.

#### ^*213*^*Bi kidney dosimetry*

The methodology as described above was used to obtain the TACs and consequently TIACs of the estimated in vivo accumulation of free ^213^Bi. Importantly, as the in vivo presence of ^225^Ac can be viewed as a nanogenerator of ^213^Bi, the decay constant (λ) of ^225^Ac was used in the dose calculation for ^213^Bi in equilibrium. Furthermore, the sum of the dose conversion factors of ^213^Bi and its daughter nuclides were applied to obtain the absorbed dose.

### Statistics

For the analysis of comparing means between two groups, significance was tested using the independent t-test with Holm-Šídák multiple comparisons correction and were considered significant at *p* < .05. Analyses were performed using GraphPad Prism version 10.0 (GraphPad Software, Inc., San Diego, CA, USA).

## Results

### ^89^Zr-FcRn(-)hG250 versus ^225^Ac-FcRn(-)hG250

#### Biodistribution

Optimization of FcRn(-)hG250 biodistribution was performed using [^111^In]In-DOTA-FcRn(-)hG250 as described in the Supplemental Material. A dose escalation study demonstrated that the mean tumour-to-liver and tumour-to-spleen ratio were highest in the 50 µg group (Supplemental Fig. 1, 2 A-B). Pre-dosing with IgG did not improve the uptake profile of radiolabelled FcRn(-)hG250 (Supplemental Fig. 2C-D, 3). Hence, subsequent experiments for ^89^Zr and ^225^Ac were performed with 50 µg FcRn(-)hG250 without pre-dosing.

**Fig. 1:**
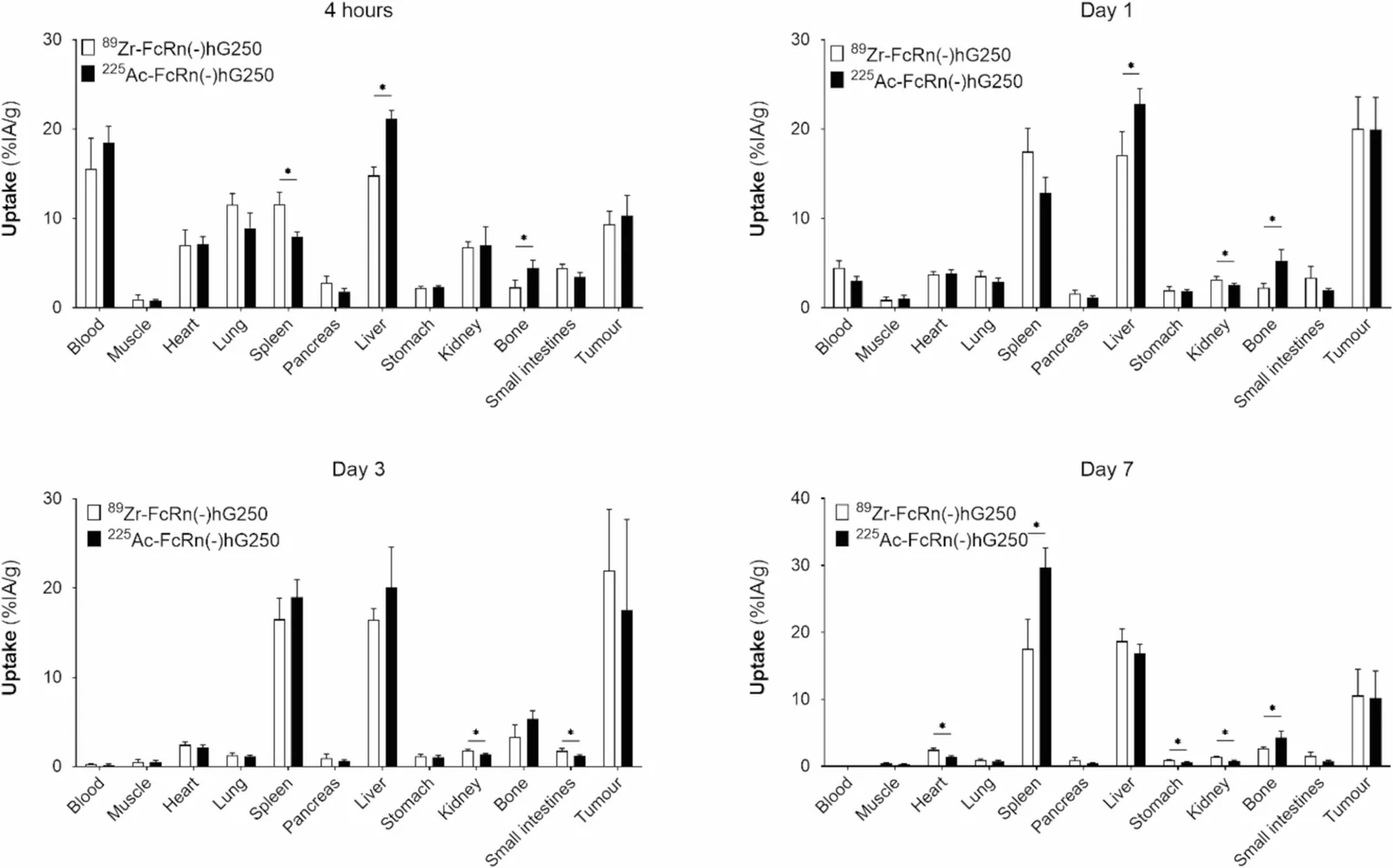
Comparison of the ex vivo biodistribution at four subsequent timepoints of ^89^Zr-FcRn(-)hG250 and ^225^Ac-FcRn(-)hG250. %IA/g = percent injected activity per gram, * *p* < .05

The tumour uptake of ^89^Zr- and ^225^Ac-FcRn(-)hG250 peaked between day 1 and 3, and decreased on day 7 p.i (day 1: 20.0 ± 3.6 and 20.0 ± 3.6%IA/g and day 7: 10.6 ± 3.9 and 10.2 ± 4.0%IA/g for ^89^Zr- and ^225^Ac-FcRn(-)hG250, respectively). The tumour uptake did not significantly differ between the ^89^Zr- and ^225^Ac-groups at any timepoint (Fig. 1).

The mean blood levels were highest at 4 h p.i. and did not differ significantly between the ^89^Zr-FcRn(-)hG250 and ^225^Ac-FcRn(-)hG250 groups (15.5 ± 3.4 vs. 18.5 ± 1.8%IA/g, respectively, *p*>.05). In both groups, blood levels rapidly decreased over time and the radioactivity in blood samples taken from mice that received ^225^Ac-FcRn(-)hG250 were below the detection level of the γ-counter for ^221^Fr/^213^Bi at 1 day p.i.

The spleen uptake was high at day 1 p.i. and remained stable over time for ^89^Zr-FcRn(-)hG250 (17.5 ± 2.6 at day 1 vs. 17.6 ± 4.4%IA/g at day 7 p.i.). In the ^225^Ac-FcRn(-)hG250 group, the relative uptake in the spleen increased over time (12.8 ± 1.8 at day 1 vs. 29.7 ± 3.0%IA/g at day 7 p.i.), resulting in a statistically significant different spleen uptake at day 1 and 7 compared to ^89^Zr-FcRn(-)hG250 (*p*<.05). Since the percentage injected activity in the spleen remained stable while the weight decreased (Supplemental Fig. 4), this finding was attributed to spleen atrophy as observed in a previous study [19].

Liver accumulation of ^225^Ac-FcRn(-)hG250 was significantly higher compared with that of ^89^Zr-FcRn(-)hG250 at 4 h and day 1 (21.2 ± 0.9 vs. 14.8 ± 0.9%IA/g at 4 h and 22.8 ± 1.7 vs. 17.1 ± 2.6 on day 1, *p*<.05).

#### Dosimetry

**Fig. 2:**
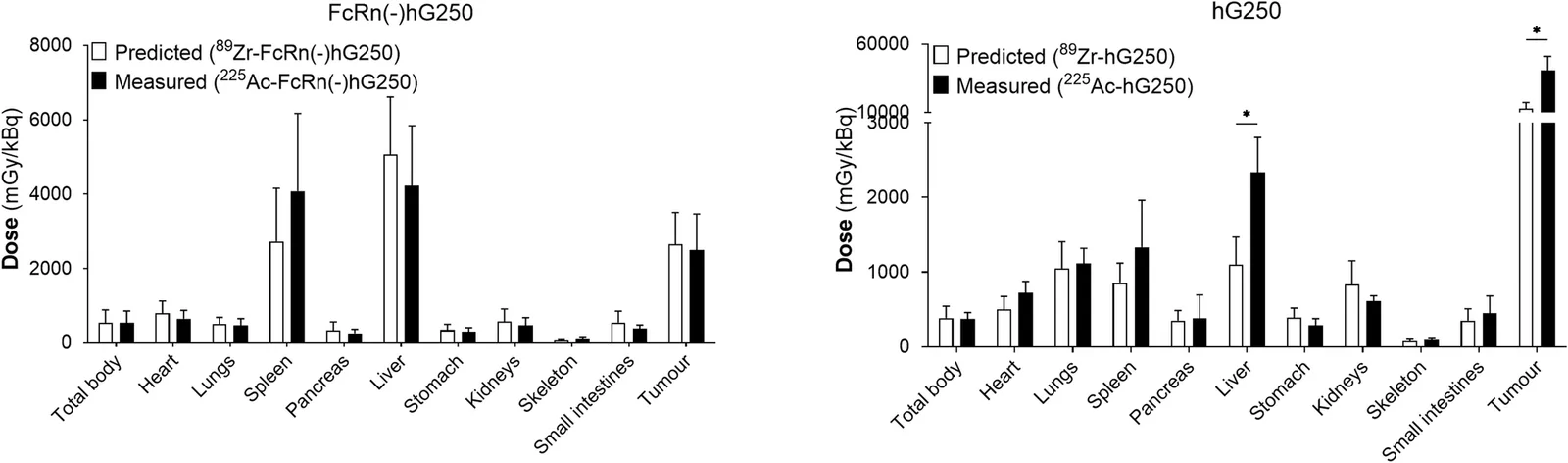
Comparison of the predicted absorbed dose, based on ^89^Zr-(FcRn(-))hG250, and measured absorbed dose, based on ^225^Ac-(FcRn(-))hG250. * *p* < .05

The absorbed doses in the specified organs were estimated for ^225^Ac, using two methods: (1) based on the biodistribution of ^89^Zr-FcRn(-)hG250 (predicted ^225^Ac-dose), or (2) based on the biodistribution of ^225^Ac-FcRn(-)hG250 (measured ^225^Ac-dose) (Fig. 2, Supplemental Table 5). The highest measured ^225^Ac-doses were calculated for the liver (4.2 ± 1.6 Gy/kBq), spleen (4.1 ± 2.1 Gy/kBq), and tumour (2.5 ± 0.97 Gy/kBq). For the tumour and all assessed organs, no statistically significant differences were found between predicted and measured absorbed doses. Although not statistically significant, the highest absolute deviation in predicted ^225^Ac-dose was observed for the liver (overestimation of 21.1 ± 10.3 Gy for a 25 kBq dose of ^225^Ac) and spleen (underestimation of 33.9 ± 24.9 Gy for a 25 kBq dose of ^225^Ac).

Given the known property of ^213^Bi to accumulate in the kidneys [27], uptake of free ^213^Bi was measured in kidneys. Accumulation of free ^213^Bi in the kidney was observed after administration of ^225^Ac-FcRn(-)hG250, with a peak at 4 h p.i. This translated to an absorbed kidney dose of 34.6 ± 5.0 Gy from ^213^Bi using an activity dose of 25 kBq ^225^Ac. Together with the kidney absorbed dose from ^225^Ac of 11.9 ± 5.2 Gy, this results in a substantially increased total absorbed kidney dose of 46.5 ± 10.2 Gy (Table 1).

**Table 1:** ^213^Bi dosimetry of ^225^Ac-hG250 and ^225^Ac-FcRn(-)hG250 in xenografted mice using OLINDA/EXM 2.1

Contribution	Absorbed dose ± RMSE			
	^213^Bi (Gy)	^225^Ac-mAb (Gy)	Total	
25 kBq of ^225^Ac-FcRn(-)hG250	34.6 ± 5.0	11.9 ± 5.2	46.5 ± 10.2	
50 kBq of ^225^Ac-hG250	95.4 ± 14.3	30.6 ± 3.6	126.0 ± 17.9	

RMSE = Root Mean Square Error. Gy = Gray. Bq = Bequerel. mAb = monoclonal antibody

### ^89^Zr-hG250 versus ^225^Ac-hG250

#### Biodistribution

**Fig. 3:**
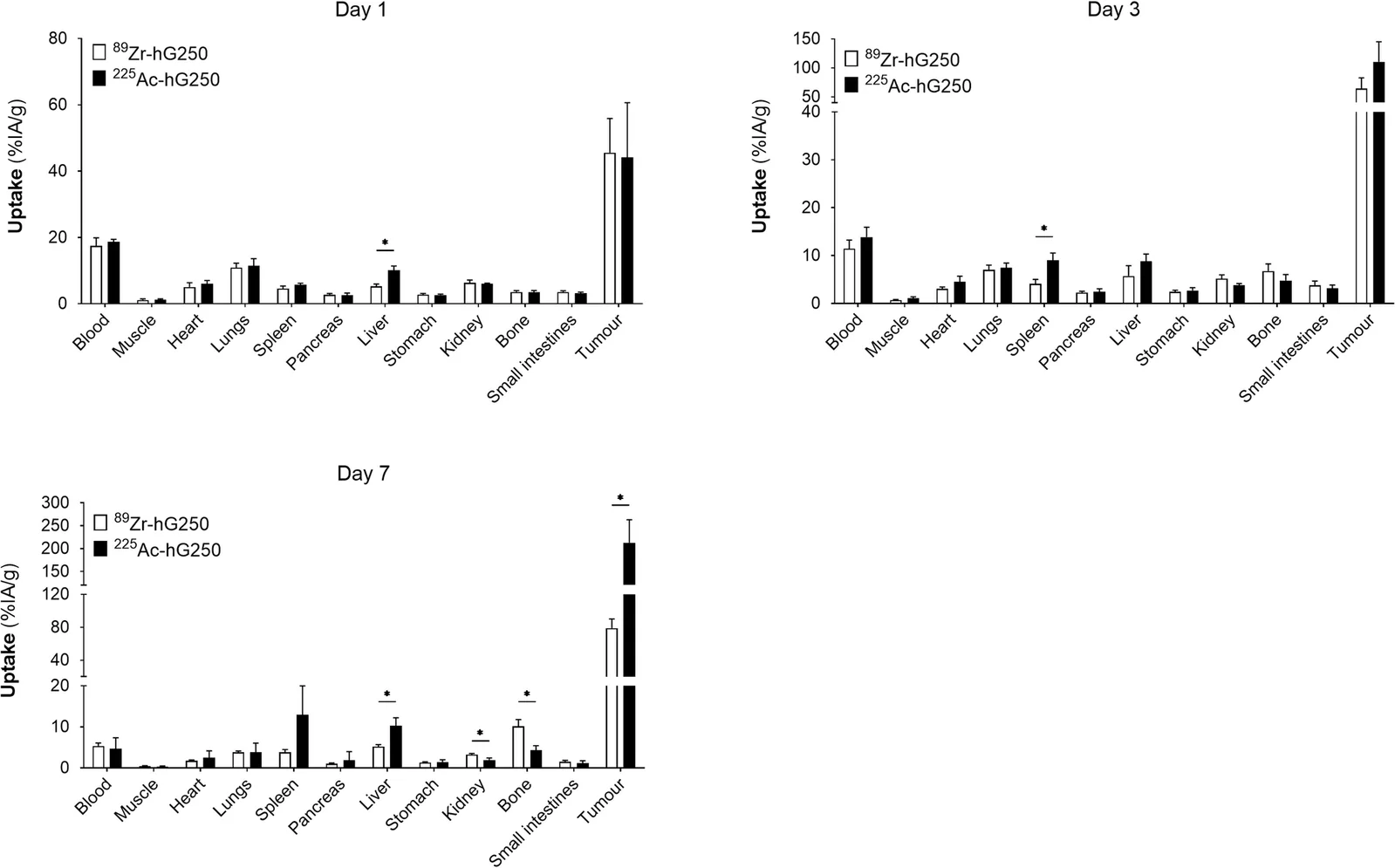
Comparison of the ex vivo biodistribution at three timepoints of ^89^Zr-hG250 and ^225^Ac-hG250 [19]. %IA/g = percent injected activity per gram, * *p* < .05

The biodistribution data of ^225^Ac-hG250 has been published previously and is presented here in comparison to ^89^Zr-hG250 [19]. The tumour uptake of ^89^Zr-hG250 and ^225^Ac-hG250 increased over time, peaked at day 7, and was significantly higher in the ^225^Ac-group compared with the ^89^Zr-group (212.8 ± 45.0 vs. 79.0 ± 9.9%IA/g, respectively, *p*<.05). In the ^89^Zr-hG250 group, bone uptake was significantly higher at day 7 (10.1 ± 1.5%IA/g vs. 4.3 ± 1.0%IA/g, respectively, *p*<.05). Liver and spleen uptake were significantly lower in ^89^Zr-hG250 compared with ^225^Ac-hG250 at day 7 (5.2 ± 0.5 vs. 10.3 ± 1.7%IA/g and 3.8 ± 0.6 vs. 13.0 ± 6.3%IA/g for the liver and spleen, respectively, *p*<.05) (Fig. 3). For spleen, this could be attributed to spleen atrophy, similar to ^225^Ac-FcRn(-)hG250 (Supplemental Fig. 4).

#### Dosimetry

The absorbed doses in the specified organs were estimated for ^225^Ac, based on the biodistribution of ^89^Zr-hG250 (predicted ^225^Ac-dose) or ^225^Ac- hG250 (measured ^225^Ac-dose) (Fig. 2, Supplemental Table 6). Highest absorbed doses were calculated in the tumour (40.9 ± 10.2 Gy/kBq), liver (2.3 ± 0.46 Gy/kBq), and spleen (1.3 ± 0.61 Gy/kBq). The largest significant deviation was found in the tumour (underestimation of 1414 ± 629 Gy for a 50 kBq dose of ^225^Ac-hG250, *p*<.05). Liver dose was significantly underestimated by 62.0 ± 24.3 Gy for a dose of 50 kBq ^225^Ac (*p*<.05). Although not statistically significant, the absorbed dose in the spleen was also slightly underestimated by 23.9 ± 13.5 Gy. Notably, tissues showing the largest deviations were those in which ^225^Ac-hG250 uptake had not yet reached a clear terminal phase at the final timepoint. Consequently, TAC extrapolations beyond the last measurement relied heavily on curve fitting, making the dose-estimates for tumour, liver, and spleen more uncertain. In the remaining organs, the predicted and measured ^225^Ac-doses were not statistically significantly different.

As published previously, accumulation of free ^213^Bi in the kidney occurred after administration of ^225^Ac-hG250 with a peak at 72 h p.i [19]. This translated to an absorbed kidney dose of 95.4 ± 14.3 Gy from free ^213^Bi using an activity dose of 50 kBq ^225^Ac. Together with the kidney absorbed dose from ^225^Ac of 30.6 ± 3.6 Gy, this resulted in a substantially increased total absorbed kidney dose of 126.0 ± 17.9 Gy (Table 1).

## Discussion

Our study demonstrates the potential and pitfalls of using ^89^Zr-FcRn(-)hG250 and ^89^Zr-hG250 as surrogate to predict the absorbed doses of ^225^Ac-FcRn(-)hG250 and ^25^Ac-hG250, respectively. As TAT moves increasingly into clinical trials, it will become crucial to conduct thorough pre-treatment dosimetry to maximize therapeutic efficacy while minimizing toxicity and unnecessary treatment of non-responding patients [1, 30–32]. Although the biodistribution of ^89^Zr- and ^225^Ac-labelled radiopharmaceuticals shared similarities, substantial differences were observed, affecting the dose predictions. Furthermore, a correction for kidney dose may be relevant as the kidney dose almost tripled for both radiopharmaceuticals when correcting for free ^213^Bi that redistributed to the kidneys.

We hypothesized that disabling the FcRn-IgG interaction would result in more favourable pharmacokinetics and a larger therapeutic window for the purpose of TRT, as it is crucial for maintaining plasma IgG-levels through recycling [24]. Indeed, our experiments show rapid plasma clearance of radiolabelled FcRn(-)hG250, accompanied with high liver and spleen uptake, which are the main sites of physiological antibody degradation [33]. This is in line with literature, e.g. ^111^In-labelled FcRn(-)anti-gD antibody showed significantly higher uptake in liver and spleen compared with the unmodified control antibody [34]. The swift plasma clearance observed for FcRn(-)hG250 resulted in a reduction of the absorbed dose in kidney compared with IgG, but was accompanied by a decrease in absorbed tumour dose, and increase in spleen and liver absorbed dose. This suggests that while modifying the FcRn-IgG interaction in hG250 may mitigate kidney exposure, it also impacts the distribution of the therapeutic agent to other organs. The substantially decreased tumour accumulation is therefore likely to compromise FcRn(-)hG250 anti-tumour efficacy. Despite our optimization efforts through dose-finding and pre-IgG dosing, FcRn(-)hG250 demonstrated less favourable biodistribution than hG250 in our studies, suggesting it is a suboptimal carrier for ^225^Ac-TAT.

Our findings show that the ^225^Ac absorbed dose can be predicted by using ^89^Zr as a surrogate when biodistribution profiles are comparable, as was shown for FcRn(-)hG250. However, for hG250 difference in biodistribution profiles were observed between the 225Ac- and 89Zr-labelled variants. Importantly, FPLC and stability analyses did not reveal clear differences between the two variants (supplemental Fig. 6), suggesting that this mismatch is unlikely to be primarily driven by major differences in radiochemical serum instability or antibody structure. The predicted absorbed dose for tumour and liver of hG250 were significantly underestimated compared with the measured absorbed dose. Notably, for the tumour, liver and spleen significantly higher uptake was observed for ^225^Ac-hG250 compared with ^89^Zr-hG250, explaining the deviation in predicted absorbed dose at least in part. This underestimation reflects a key limitation of using only three timepoints for dosimetry, particularly when the terminal phase of uptake is not captured. In our study, tumour and spleen activity continued to rise on day 7, and liver uptake showed no evidence of washout. As a result, the necessary extrapolation into later unmeasured time intervals increased the uncertainty of the curve fits and reduced the accuracy of the ^225^Ac doses in these tissues. Based on the biological half-life of the antibodies determined from the TACs of whole-body uptake (supplemental Fig. 7), the tumour dose calculations for ^89^Zr-hG250 and ^225^Ac-hG250 derived from TACs extrapolated to infinity may overestimate the absorbed dose by approximately 26% and 59%, respectively (TIACs of 14.5 and 25.6 h when accounting for biological half-life, versus 19.6 and 62.3 h based on the reported extrapolations). Therefore, inclusion of a later timepoint is recommended for future studies, for example > 14 days for ^225^Ac-labelled antibodies and 10 days for ^89^Zr-labelled antibodies (limited by physical half-life).

Although inclusion of later timepoints may therefore reduce the extent of the observed discrepancies between ^225^Ac-hG250 and ^89^Zr-hG250 in terms of tumour-absorbed dose, we do not expect this to substantially alter the overall conclusions of the study. The differences in uptake between the two radiolabelled variants in both tumour and liver are already clearly evident on day 7 and would therefore continue to contribute to differences in absorbed dose estimates. Moreover, liver uptake follows a saturating profile in contrast to the near-linear increase observed for ^225^Ac-hG250 in the tumour (supplemental Fig. 8). Based on these kinetics, inclusion of later timepoints would be expected to have only a limited impact on the estimated liver dose, further supporting the robustness of the observed differences.

Accurate prediction of tumour absorbed dose is crucial, as the clinical outcome of TRT likely depends on the attainable tumour dose [35, 36]. Differences in tumour uptake between radiopharmaceuticals labelled with imaging versus therapeutic radionuclides may stem from treatment-induced alterations in the tumour environment. For example, irradiation has been suggested to augment the enhanced permeability and retention (EPR) effect of antibodies in tumour tissue, potentially leading to a more favourable biodistribution profile [19, 37]. This aligns with our observations for hG250, although this was not observed for FcRn(-)hG250. This may be explained by the lower total tumour uptake and the use of 25 kBq instead of 50 kBq of ^225^Ac. Importantly, we observed no significant differences in tumour weights between ^225^Ac- and ^89^Zr-labelled compounds at any timepoint for both agents (supplemental Fig. 4), indicating that the observed differences in tumour uptake cannot be attributed to variations in tumour size. However, further research is needed to determine whether therapeutic radionuclides systematically influence tumour uptake or whether this is a model- or radioisotope-specific phenomenon. Moreover, to what extent this affects other targeting agents should be evaluated to better understand the clinical translatability of our observation.

Beyond tumour dose, accurate prediction of radiation dose in healthy organs is critical to determine the risk of toxicity. In this study, the theranostic pair of the radioactive transition metal ^89^Zr and the actinoid ^225^Ac was chelated with DFO and DOTA, respectively. These radionuclide-chelator combinations possess intrinsically different stability and in vivo redistribution of released radionuclides. The ^89^Zr-DFO complex may dissociate, releasing bone-seeking ^89^Zr [38], whereas free ^225^Ac will accumulate primarily in the liver and, to a lesser extent, bone [39]. Although the uptake of ^89^Zr-hG250 was indeed significantly higher in bone compared with ^225^Ac-hG250, this did not significantly influence the absorbed dose prediction. Moreover, bone deposition of ^89^Zr has not been routinely observed in clinical studies thus far and may be radiopharmaceutical-dependent [40]. On the other hand, if ^225^Ac dissociates from the DOTA complex, it will accumulate in the liver, which may explain the underestimation of absorbed dose to the liver when using ^89^Zr-hG250 biodistribution for predictive dosimetry. Following this, determining the stability of ^89^Zr-DFO and ^225^Ac-DOTA in specific radiopharmaceuticals is important in future studies, and a different chelator with superior in vivo stability, such as DFO* or HOPO for ^89^Zr [40] or H2macropa for ^225^Ac [41], may overcome this issue [42, 43]. This will help clarify whether the observed differences between ^89^Zr and ^225^Ac can be attributed to radiochemical differences or by their different biological effects.

We observed significant kidney accumulation of free ^213^Bi, most likely caused by recoil of the daughter radionuclide following alpha decay, and is in line with a previous study [27]. The redistribution contributed substantially to the renal absorbed dose, making it approximately three times higher than when considering only ^225^Ac. As a result, accurate kidney dose estimation based on ^89^Zr-labelled counterparts remains challenging, and a correction factor may be necessary for dosimetry calculations. However, the clinical significance of this effect remains to be determined, since all current observations stem from preclinical models. Importantly, the interpretation of these findings is further complicated by the simplified dosimetric approaches commonly used in preclinical studies, including the present work, in which the kidney is treated as a homogeneous organ. Both antibody uptake and redistribution of free ^213^Bi are expected to be predominantly localized in the renal cortex [44]. Therefore, these conventional organ-level dose estimates likely underestimate the absorbed dose to the cortex while overestimating exposure in other renal compartments. In addition, anatomical differences between mouse and human kidneys, particularly with respect to organ size relative to the short path length of ^213^Bi, may substantially influence dose distribution and biological effects. Consequently, the development of quantitative correction factors based on preclinical data remains challenging. Furthermore, this phenomenon is likely radiopharmaceutical-dependent, influenced by factors such as chelator stability [45], binding and internalization properties, and circulation time. Future studies should systematically assess these variables to refine kidney dosimetry and evaluate the potential risks for translation towards a clinical setting. Notably, current studies, mainly using fast-clearing agents (peptides and small molecules) report limited or no ^225^Ac-nephrotoxicity [46].

Although not statistically significant, we observed an inaccuracy of approximately 30% in the prediction of spleen dose for both ^225^Ac-labelled radiopharmaceuticals. This could be attributed to the subacute toxic effect of high doses of ^225^Ac-labelled antibody on the spleen of mice, leading to a substantial decrease in spleen mass (supplemental Fig. 5), which is in line with other preclinical literature [47–49]. As with nephrotoxicity, reports of splenic toxicity in clinical settings are currently absent, and therefore the clinical consequence of this remains to be determined. The therapeutic window of TAT is likely to be different between mice and humans, as the short range of alpha particles may lead to different dose distributions and toxicity thresholds depending on organ size. Nevertheless, these results warrant further investigation with lower doses of ^225^Ac to understand which part of the described differences can be attributed to biological effects of ^225^Ac that are not observed with ^89^Zr.

For proper interpretation of our findings, the following limitations in the study design need to be considered: first, the data for the hG250 and FcRn(-)hG250 were obtained in separate experiments, therefore a head-to-head comparison of radiolabelled hG250 and FcRn(-)hG250 might be influenced by interexperimental differences. Second, the dosimetry is based on the harvested organs and thus accurate assessment of the non-studied organs has not been performed. For example, we did not evaluate bone marrow, one of the most dose-limiting organs in β-TRT, because the activity levels in the very small quantities of bone marrow could not be reliably measured [50]. Third, the applied robust kidney dosimetry model considers homogenous distribution of activity, which is not the case for particles with a short range in tissue, such as alpha-particles. Therefore, it becomes relevant to assess the distribution within the kidney, but potentially also within the tumour when the intratumoural dose distribution is heterogeneous [51], as is likely in a clinical setting. Finally, the dosimetry estimates for ^225^Ac-hG250 were derived from only three timepoints, and for tumour, liver, and spleen the uptake kinetics had not yet reached a peak or clearance phase at the final measurement. This required extrapolation from incomplete TACs, reducing confidence in dose predictions for these tissues. Inclusion of later timepoints capturing the clearance phase would likely reduce the area under the TACs, potentially narrowing the observed discrepancies with ^89^Zr-derived dose predictions.

## Conclusion

This study demonstrates that the theranostic pair of ^89^Zr/^225^Ac showed variability in the prediction of organ and tumour doses for two different CAIX-targeting antibodies in a ccRCC mouse model. While tumour absorbed dose prediction was accurate for FcRn(-)hG250, significant differences in uptake between ^89^Zr-hG250 and ^225^Ac-hG250 led to inaccurate absorbed dose predictions for hG250 in tumour and liver. Furthermore, kidney dose estimates are likely underestimated due to redistribution of ^213^Bi due to the recoil effect, necessitating corrective measures. Additional translational and clinical studies are essential to understand how we can use ^89^Zr-PET to predict ^225^Ac tumour and organ doses in the clinical setting.

## Supplementary information

Below is the link to the electronic supplementary material.

Supplementary Material 1 (DOCX 5.05 MB)

## Data Availability

The datasets generated during and/or analysed during the current study are partly published as supplemental data, the remaining datasets are available from the corresponding author on reasonable request.
