# Supplementary material for: Predicting tumour and organ dose in α-radiopharmaceutical therapy: a 89Zr-based dosimetric approach for 225Ac
Source: Eur J Nucl Med Mol Imaging. 2026 Jul 15;53(12):7082–92. doi: 10.1007/s00259-026-08076-x (PMC13633313; doi:10.1007/s00259-026-08076-x)
Supplement: Supplementary file 1 [file 259_2026_8076_MOESM1_ESM.docx]

**Supplemental Data**

**Materials & Methods**

**Conjugation, Labeling & Quality Control**

FcRn(-)hG250 and hG250 were conjugated with N-succinyldesferrioxamine-B-tetrafluorphenol (N-SucDf-TFP/DFO) (VU Medical Centre, Amsterdam, The Netherlands) as described previously [1].

FcRn(-)hG250: 50.3 MBq of ^89^Zr-oxalate was added to 19 µl of Na_2_CO_3_ 2M and incubated for 3 minutes. Subsequently, HEPES buffer 0.5M pH 7.2 and 1100 µg FcRn(-)hG250-DFO were added and incubated for 60 minutes at room temperature. Radiochemical purity was determined by instant thin layer chromatography (ITLC), using ITLC silica gel strips (Agilent Technologies, Santa Clara, CA, USA), and 0.1 M of citrate buffer (pH 6.0) analyzed using the Typhoon FLA 7000 (GE Healthcare, Chicago, IL, USA), and was >95%. After incubation PBS/BSA 0.5% was added, resulting in a final concentration of 11.4 MBq/mL [^89^Zr]Zr-DFO-FcRn(-)hG250 (^89^Zr-FcRn(-)hG250, specific activity 0.045 MBq/µg).

hG250: 165 MBq of ^89^Zr-oxalate was added to 90 µl of Na_2_CO3 2M and incubated for 3 minutes. Subsequently, HEPES buffer 0.5M pH 7.2 and 500 µg hG250-DFO were added and incubated for 60 minutes at room temperature. Labelling efficiency was determined by ITLC and was 22%. Labelling mixture was purified by PD10 purification eluted with PBS/BSA 0.5% (radiochemical purity: 99%). After purification hG250 and PBS/BSA 0.5% were added, resulting in a final concentration of 5.9 MBq/mL [^89^Zr]Zr-DFO-hG250 (^89^Zr-hG250, specific activity 0.073 MBq/µg).

hG250 and FcRn(-)hG250 were conjugated with S-2-(4-Isothiocyanatobenzyl)-1,4,7,10-tetraazacyclododecane tetraacetic acid (p-SCN-Bn-DOTA, Macrocyclics™, Plano, TX, US) as described previously [2].
FcRn(-)hG250: 923 kBq of ^225^Ac-chloride (33.4 uL) was added to 2472 µl of TRIS 0.1M pH 9 buffer and 1620 µg FcRn(-)hG250-DOTA and incubated for 60 minutes at 37°C. Radiochemical purity was determined directly after incubation by ITLC analyzed using the Typhoon FLA 7000 (GE Healthcare, Chicago, IL, USA) and was 97%. After incubation EDTA 50 mM; PBS 10x; HCl 2M, PBS/BSA 0.5% was added, resulting in a final concentration of 144 kBq/mL [^225^Ac]Ac-DOTA-FcRn(-)hG250 (^225^Ac-FcRn(-)hG250, specific activity 0.55 kBq/µg).

hG250: 3.1 MBq of ^225^Ac-chloride (16.2 uL) was added to 1200 µl of TRIS 0.1M pH 9 buffer and 1000 ug hG250-DOTA and incubated for 60 minutes at 37°C. Radiochemical purity was determined by ITLC and was 99%. After incubation EDTA 50 mM; PBS 10x; PBS/BSA 0.5% and hG250 was added, resulting in a final concentration of 250 kBq/mL [^225^Ac]Ac-DOTA-hG250 (^225^Ac-hG250, specific activity 3.1 kBq/µg).

FcRn(-)hG250 was conjugated with S-2-(4-Isothiocyanatobenzyl)-1,4,7,10-tetraazacyclododecane tetraacetic acid (p-SCN-Bn-DOTA, Macrocyclics™, Plano, TX, US) and labelled with ^111^In as described previously [2]. Radiochemical purity was determined by instant thin layer chromatography (ITLC), using ITLC silica gel strips (Agilent Technologies, Santa Clara, CA, USA), and 0.1 M of citrate buffer (pH 6.0). Labeling efficiency exceeded 95%. The immunoreactive fraction of all labelled antibodies was performed using SK-RC-52 cells as described by Lindmo et al. and exceeded 80%.

Size-exclusion fast protein liquid chromatography (SEC-FPLC) was performed to assess the radiochemical profile and stability of the constructs. Radiolabelings were performed at specific activity as described above and were injected to FPLC directly after. Injection solutions with concentrations as desribed above were prepared and left for approximately 2 hours at RT, before injecting on FPLC. To simulate *in vivo* stability, constructs were incubated in 50% normal mouse serum (Bodinco, 5097) at 125 kBq/mL for ^225^Ac-labelled constructs and 5.9 MBq/mL for ^89^Zr-labelled constructs. Analyses were conducted using a Äkta Purifier 10 FPLC system (GE Healthcare) equippped with a Superdex 200 10/300 GL column. The mobile phase consisted of PBS, pH 7.4, applied under isocratic conditions at a flow rate of 0.6 mL/min. For ^225^Ac-labelled constructs, the FPLC eluent was collected in 0.5 mL fractions using an automated fraction collector (Äkta Frac-920, GE Healthcare). Collected fractions were measured using a γ-counter (1480 Wizard 3; LKB/Wallace, Perkin Elmer, Boston, MA) using ^221^Fr as a surrogate for ^225^Ac after > 1 h after dissection (at ^225^Ac-^221^Fr equilibrium). For ^89^Zr-labelled constructs, radioactivity was monitored using a radiodetector (GABI*, Elysia-raytest) coupled directly to the FPLC system, allowing continuous detection.

**Optimization of FcRn(-)hG250**

*Protein dose escalation*

Twenty female BALB/cAnNRj-Foxn1^nu/nu^ mice bearing SK-RC-52 xenografts were injected with 10, 30, 50 or 100 µg ^111^In-FcRn(-)hG250. After 3 days, mice were sacrificed and dissected to perform *ex vivo* biodistribution studies. The tumour and normal tissues (blood, muscle, heart, lungs, spleen, pancreas, liver, stomach, kidneys, bone, small intestine without contents) were harvested, weighed and counted in a γ-counter (1480 Wizard 3; LKB/Wallace, Perkin Elmer, Boston, MA). To correct for radioactive decay, three aliquots containing 1% of the injected dose were counted simultaneously. The activity in samples was expressed as percent injected dose per gram tissue (%IA/g) and reported as mean ± standard deviation.

*Pre-dosing with immunoglobulin*

Ten SK-RC-52-tumour bearing mice were divided in two groups. One group was injected intraperitoneally with 2.5 mg human immunoglobulin (Nanogam, Sanquin Plasma Products, Netherlands), two days prior to intravenous administration of ^111^In-FcRn(-)hG250. After 3 days, *ex vivo* biodistribution was performed as described previously.

**Biodistribution of ^213^Bi in kidney**

The total ^213^Bi activity in kidneys at time of sacrifice (t=0) was determined by using the function:

$$A_{Bi}\left( t \right)=\frac{\lambda_{At} \lambda_{Fr} \lambda_{Bi}}{\lambda_{Fr}-\lambda_{Ac}}A_{Ac}(0)\left\{ \frac{e^{-\lambda_{Ac}t}-e^{-\lambda_{Bi}t}}{\left( \lambda_{Bi}-\lambda_{Ac} \right)\left( \lambda_{At}-\lambda_{Ac} \right)}-\frac{e^{-\lambda_{At}t}-e^{-\lambda_{Bi}t}}{\left( \lambda_{Bi}-\lambda_{At} \right)\left( \lambda_{At}-\lambda_{Ac} \right)}-\frac{e^{-\lambda_{Fr}t}-e^{-\lambda_{Bi}t}}{\left( \lambda_{Bi}-\lambda_{Fr} \right)\left( \lambda_{At}-\lambda_{Fr} \right)}+\frac{e^{-\lambda_{At}t}-e^{-\lambda_{Bi}t}}{\left( \lambda_{Bi}-\lambda_{At} \right)\left( \lambda_{At}-\lambda_{Fr} \right)} \right\}$$

, with *λ_Ac_, λ_Fr_, λ_At_* and *λ_Bi_* the decay constants for ^225^Ac, ^221^Fr, ^207^At and ^213^Bi, respectively. This equation represents the ^213^Bi activity being in equilibrium with the initial ^225^Ac activity, whereas the free ^213^Bi activity follows the exponential decay function: $A_{Bi}\left( t \right)=A_{Bi}(0)e^{-\lambda_{Bi}t}$. Summing the equilibrium and free bismuth activity and grouping the separate decay functions the following overall ^225^Ac / ^213^Bi decay function was defined in Graphpad Prism:

$$\boldsymbol{Y}\left( \boldsymbol{t} \right)\boldsymbol{=A\times}\left( \boldsymbol{1.0035}\boldsymbol{e}^{\boldsymbol{-}\boldsymbol{\lambda}_{\boldsymbol{Ac}}\boldsymbol{t}}\boldsymbol{+0.1205}\boldsymbol{e}^{\boldsymbol{-}\boldsymbol{\lambda}_{\boldsymbol{Fr}}\boldsymbol{t}}\boldsymbol{+1.1812}\boldsymbol{E-5}\boldsymbol{e}^{\boldsymbol{-}\boldsymbol{\lambda}_{\boldsymbol{At}}\boldsymbol{t}}\boldsymbol{-1.1240}\boldsymbol{e}^{\boldsymbol{-}\boldsymbol{\lambda}_{\boldsymbol{Bi}}\boldsymbol{t}} \right)\boldsymbol{+B\times}\boldsymbol{e}^{\boldsymbol{-}\boldsymbol{\lambda}_{\boldsymbol{Bi}}\boldsymbol{t}}$$

with A the initial ^225^Ac activity and B the initial free 213Bi activity. Fitting of the time-activity data was performed using this function with A and B as free parameters.

**Statistics**

For the analysis of comparing means between two groups, significance was tested using the independent t-test with Holm-Šídák multiple comparisons correction and were considered significant at p <.05. In the analysis of comparing means between more than two groups, One-way ANOVA with Tukey multiple comparison correction was used for each organ, followed by Bonferroni correction. Analyses were performed using GraphPad Prism version 10.0 (GraphPad Software, Inc., San Diego, CA, USA).

**Results**

**Optimization of FcRn(-)hG250**

*Protein dose escalation*

^111^In-FcRn(-)hG250 showed high uptake in CAIX-expressing tumours (Table 1-4). The tumour uptake of 30, 50, and 100 µg ^111^In-FcRn(-)hG250 did not differ significantly at 3 days p.i. (17.5 ± 7.4, 21.5 ± 8.7, 19.3 ± 7.1 %IA/g, respectively p>.05)(Fig. 1). However, tumour accumulation was significantly lower in the 10 µg group compared with the 50 µg group (9.1 ± 3.5 %IA/g vs. 21.5 ± 8.7 %IA/g, p<.05).

Other organs that demonstrated high tracer uptake were spleen, liver, and bone marrow (Fig. 1). There was no significant difference in the liver uptake between the groups (20.8 ± 2.7, 22.3 ± 3.4, 19.0 ± 3.9, 22.2 ± 3.5 %IA/g for 10, 30, 50, and 100 µg, respectively, p>.05). The mean tumour- to-liver and tumour-to-spleen ratio were highest in the 50 µg group (1.2 and 1.8, respectively) (Fig. 2A+B), although not tested for significance due to lack of power in the study design.

*Pre-dose IgG*

In the protein dose escalation study, we observed a high relative uptake in non-target tissue, mainly the spleen and liver. To rule out that a lack of endogenous immunoglobulin in these relatively young mice contributed to this biodistribution profile, we performed a pre-dose IgG study. The tumour, spleen and liver uptake was not affected by a pre-dose of 2.5 mg of human immunoglobulin in our study. Due to the small sample size no statistics were performed in these groups (Fig. 2). Furthermore, tumour-to-liver and tumour-to-spleen ratio in both groups were similar (Fig. 2C+D).

Based on the protein dose escalation and pre-dose studies, subsequent studies were performed with a protein dose of 50 µg without pre-dosing.

**Supplemental Figures.**

Supplemental figure 1. *Ex-vivo* biodistribution data of mice that received 10, 30, 50, or 100 µg of ^111^In-FcRn(-)hG250. %IA/g = percent injected activity per gram. * p < 0.0042 (Bonferroni-corrected alpha).

Supplemental figure 2. A-B: Tumour to organ ratios in mice that received 10, 30, 50 or 100 µg of ^111^In-DOTA-FcRn(-)hG250. C-D: Tumour to organs ratios of immunoglobulin predosed mice compared to control mice that received 50 µg ^111^In-DOTA-FcRn(-)hG250.

Supplemental figure 3. *Ex-vivo* biodistribution data of mice that received 1 MBq of ^111^In-FcRn(-)hG250 with or without IgG pre-dosing. %IA/g = percent injected activity per gram.

Supplemental figure 4. Tumour weights of the different groups in the FcRn(-)hG250 and hG250 biodistribution experiments with ^89^Zr and ^225^Ac. nd = not a discovery

Supplemental figure 5. Spleen weights of the different groups in the FcRn(-)hG250 and hG250 biodistribution experiments with ^89^Zr and ^225^Ac. * = significant discovery, nd = not a discovery

Supplemental figure 6. SEC-FLPC results for [89Zr]Zr-DFO-hG250 and [225Ac]Ac-DOTA-hG250 directly after radiolabeling, 2h after preparing injetion solution, and 1, 4 and 6 or 7 days after preparing serum solution.

Supplemental figure 7. Whole-body time-activity curves, considering the acitivity measured in each organ multiplied by organ weight. Curves were fitted by single exponential curves to determine biological half-lifes of 295 h (CI: 197-559 h) for ^89^Zr-DFO-hG250 and 211 h (CI: 166-184 h) for ^225^Ac-DOTA-hG250

Supplemental figure 8. Tumor and liver time-acitivity curves.

**Supplemental Tables.**

Supplemental table 1. Biodistribution data of mice that received 10, 30, 50, or 100 µg ^111^In-DOTA-FcRn(-)hG250, reported are the percent injected activity per gram (%IA/g) and netto weight of the organs (NWO). (Data of Supplemental Fig. 1)

|  | **Treatment A (10 µg protein dose)** | | | | | | | | | |
| --- | --- | --- | --- | --- | --- | --- | --- | --- | --- | --- |
|  | Mouse 1 | | Mouse 2 | | Mouse 3 | | Mouse 4 | | Mouse 5 | |
|  | %IA/g | NWO (g) | %IA/g | NWO (g) | %IA/g | NWO (g) | %IA/g | NWO (g) | %IA/g | NWO (g) |
| Blood | 0.037 | 0.7905 | 0.131 | 0.1739 | 0.161 | 0.4853 | 0.173 | 0.7854 | 0.026 | 0.7035 |
| Muscle | 0.331 | 0.1091 | 0.196 | 0.1243 | 0.214 | 0.1496 | 0.231 | 0.1605 | 0.368 | 0.1337 |
| Heart | 2.273 | 0.1275 | 0.956 | 0.1253 | 1.154 | 0.1228 | 1.335 | 0.133 | 1.426 | 0.1237 |
| Lung | 0.853 | 0.1242 | 0.504 | 0.1619 | 0.458 | 0.1407 | 0.773 | 0.1963 | 0.570 | 0.1326 |
| Spleen | 13.010 | 0.0834 | 8.592 | 0.0864 | 8.118 | 0.1122 | 9.075 | 0.107 | 7.164 | 0.1195 |
| Pancreas | 1.137 | 0.2314 | 0.520 | 0.0924 | 0.476 | 0.2101 | 0.694 | 0.0899 | 0.369 | 0.1958 |
| Liver | 19.497 | 0.3105 | 17.057 | 0.3542 | 22.710 | 0.7631 | 23.895 | 0.3429 | 20.916 | 0.3805 |
| Stomach | 0.731 | 0.1022 | 0.400 | 0.1262 | 0.447 | 0.1189 | 0.685 | 0.1313 | 0.481 | 0.1202 |
| Kidney | 1.826 | 0.3211 | 1.462 | 0.4033 | 1.172 | 0.3382 | 1.655 | 0.3429 | 1.325 | 0.3778 |
| Bone | 1.527 | 0.0308 | 0.869 | 0.0259 | 1.307 | 0.0175 | 1.080 | 0.0322 | 1.110 | 0.0278 |
| Small intestines | 1.270 | 0.1853 | 1.009 | 0.1259 | 0.812 | 0.0803 | 1.097 | 0.1397 | 1.096 | 0.0962 |
| Tumour | 6.616 | 0.0252 | 8.244 | 0.1537 | 7.659 | 0.1122 | 15.331 | 0.05 | 7.713 | 0.1308 |
|  | **Treatment B (30 µg protein dose)** | | | | | | | | | |
|  | Mouse 1 | | Mouse 2 | | Mouse 3 | | Mouse 4 | | Mouse 5 | |
|  | %IA/g | NWO (g) | %IA/g | NWO (g) | %IA/g | NWO (g) | %IA/g | NWO (g) | %IA/g | NWO (g) |
| Blood | 0.100 | 0.297 | 0.052 | 0.7191 | 0.043 | 0.4287 | 0.080 | 0.7387 | 0.038 | 0.791 |
| Muscle | 0.290 | 0.0811 | 0.361 | 0.1244 | 0.382 | 0.1314 | 0.385 | 0.1175 | 0.376 | 0.0905 |
| Heart | 1.623 | 0.1169 | 2.422 | 0.1272 | 2.561 | 0.1054 | 2.059 | 0.132 | 2.338 | 0.1362 |
| Lung | 0.676 | 0.2038 | 0.706 | 0.2164 | 0.847 | 0.1707 | 0.707 | 0.1269 | 0.809 | 0.1451 |
| Spleen | 13.936 | 0.0835 | 13.527 | 0.1068 | 17.665 | 0.0789 | 11.135 | 0.0949 | 12.369 | 0.1094 |
| Pancreas | 0.725 | 0.0964 | 0.697 | 0.1644 | 0.644 | 0.1646 | 2.290 | 0.1885 | 1.005 | 0.1688 |
| Liver | 17.412 | 0.3789 | 24.212 | 0.7102 | 20.808 | 0.3008 | 26.211 | 0.7967 | 22.611 | 0.4334 |
| Stomach | 0.885 | 0.0897 | 0.700 | 0.1216 | 0.947 | 0.0977 | 0.789 | 0.1053 | 0.956 | 0.1189 |
| Kidney | 1.534 | 0.359 | 1.899 | 0.3247 | 1.751 | 0.3141 | 1.872 | 0.3011 | 1.856 | 0.3311 |
| Bone | 1.594 | 0.0204 | 2.120 | 0.0175 | 1.407 | 0.0287 | 2.495 | 0.0161 | 2.196 | 0.0165 |
| Small intestines | 1.369 | 0.0925 | 0.984 | 0.1362 | 1.948 | 0.1229 | 1.330 | 0.2029 | 1.482 | 0.1658 |
| Tumour | 15.161 | 0.0682 | 30.655 | 0.0628 | 14.388 | 0.1754 | 12.873 | 0.0759 | 14.263 | 0.1016 |
|  | **Treatment C (50 µg protein dose)** | | | | | | | | | |
|  | Mouse 1 | | Mouse 2 | | Mouse 3 | | Mouse 4 | | Mouse 5 | |
|  | %IA/g | NWO (g) | %IA/g | NWO (g) | %IA/g | NWO (g) | %IA/g | NWO (g) | %IA/g | NWO (g) |
| Blood | 0.049 | 0.587 | 0.097 | 0.587 | 0.138 | 0.3401 | 0.060 | 0.4918 | 0.097 | 0.4561 |
| Muscle | 0.789 | 0.0411 | 0.791 | 0.0411 | 0.315 | 0.1166 | 0.311 | 0.139 | 0.614 | 0.1403 |
| Heart | 1.338 | 0.1771 | 2.374 | 0.1771 | 2.338 | 0.1469 | 2.203 | 0.128 | 2.063 | 0.1478 |
| Lung | 0.715 | 0.1492 | 0.831 | 0.1492 | 0.659 | 0.1158 | 0.839 | 0.1184 | 0.763 | 0.1783 |
| Spleen | 8.700 | 0.1389 | 11.360 | 0.1389 | 16.252 | 0.0688 | 11.997 | 0.0998 | 11.970 | 0.0976 |
| Pancreas | 0.548 | 0.1748 | 0.778 | 0.1748 | 1.184 | 0.0644 | 0.640 | 0.224 | 0.619 | 0.1871 |
| Liver | 16.212 | 0.5323 | 17.215 | 0.5323 | 25.942 | 0.3451 | 17.875 | 0.7269 | 17.994 | 0.336 |
| Stomach | 0.861 | 0.1188 | 0.990 | 0.1188 | 0.964 | 0.0961 | 0.790 | 0.1041 | 1.157 | 0.1314 |
| Kidney | 2.120 | 0.3623 | 1.849 | 0.3623 | 1.850 | 0.3701 | 1.713 | 0.3328 | 1.805 | 0.3967 |
| Bone | 1.217 | 0.0147 | 2.197 | 0.0147 | 2.193 | 0.028 | 1.836 | 0.0178 | 1.998 | 0.0242 |
| Small intestines | 1.337 | 0.1878 | 2.519 | 0.1878 | 1.799 | 0.0885 | 1.670 | 0.1106 | 1.782 | 0.1017 |
| Tumour | 12.710 | 0.0436 | 18.986 | 0.0436 | 18.448 | 0.1633 | 35.872 | 0.0389 | 21.329 | 0.1156 |
|  | **Treatment D (100 µg protein dose)** | | | | | | | | | |
|  | Mouse 1 | | Mouse 2 | | Mouse 3 | | Mouse 4 | | Mouse 5 | |
|  | %IA/g | NWO (g) | %IA/g | NWO (g) | %IA/g | NWO (g) | %IA/g | NWO (g) | %IA/g | NWO (g) |
| Blood | 0.037 | 0.7622 | 0.073 | 0.4291 | 0.062 | 0.5302 | 0.032 | 0.8747 | 0.031 | 0.7355 |
| Muscle | 0.703 | 0.1529 | 0.850 | 0.156 | 0.638 | 0.1114 | 0.417 | 0.1593 | 0.382 | 0.1335 |
| Heart | 2.135 | 0.1197 | 1.662 | 0.1693 | 2.167 | 0.145 | 2.218 | 0.1316 | 1.904 | 0.1372 |
| Lung | 0.710 | 0.1305 | 0.925 | 0.1356 | 0.847 | 0.145 | 0.904 | 0.1421 | 0.630 | 0.1583 |
| Spleen | 12.084 | 0.1151 | 14.401 | 0.1038 | 17.834 | 0.077 | 10.625 | 0.1033 | 10.115 | 0.1139 |
| Pancreas | 0.871 | 0.2172 | 1.352 | 0.1261 | 0.890 | 0.2544 | 1.189 | 0.2613 | 3.628 | 0.1081 |
| Liver | 27.099 | 0.3224 | 18.525 | 0.3699 | 19.618 | 0.3278 | 24.114 | 0.3403 | 21.869 | 0.3469 |
| Stomach | 0.729 | 0.1248 | 1.172 | 0.0915 | 0.996 | 0.1097 | 0.907 | 0.1075 | 0.829 | 0.1128 |
| Kidney | 1.683 | 0.3347 | 2.053 | 0.3255 | 2.069 | 0.3148 | 1.689 | 0.3797 | 1.722 | 0.3652 |
| Bone | 1.016 | 0.027 | 2.652 | 0.0265 | 2.035 | 0.0185 | 1.292 | 0.0277 | 0.879 | 0.0236 |
| Small intestines | 1.511 | 0.1591 | 1.839 | 0.1395 | 1.733 | 0.1351 | 1.156 | 0.1428 | 1.125 | 0.1385 |
| Tumour | 16.563 | 0.0789 | 25.811 | 0.0671 | 27.804 | 0.0745 | 13.240 | 0.1379 | 12.922 | 0.0809 |

Supplemental table 2. Biodistribution data of mice that received 50 µg ^111^In-DOTA-FcRn(-)hG250 and were predosed with 2.5 milligram of immunoglobulin or not, reported are the percent injected activity per gram (%IA/g) and netto weight of the organs (NWO). (Data of Supplemental fig. 3).

|  | **Treatment A (pre-dosed with immunoglobulin)** | | | | | | | | | | | | | | | | | |
| --- | --- | --- | --- | --- | --- | --- | --- | --- | --- | --- | --- | --- | --- | --- | --- | --- | --- | --- |
|  | Mouse 1 | | Mouse 2 | | | | Mouse 3 | | | | Mouse 4 | | | | Mouse 5 | | | |
|  | %IA/g | NWO (g) | %IA/g | | NWO (g) | | %IA/g | | NWO (g) | | %IA/g | | NWO (g) | | %IA/g | | NWO (g) | |
| Blood | 0.054 | 0.862 | 0.031 | | 0.431 | | 0.063 | | 1.080 | | 0.034 | | 0.672 | | 0.048 | | 0.773 | |
| Muscle | 0.409 | 0.064 | 0.216 | | 0.107 | | 0.415 | | 0.037 | | 0.239 | | 0.064 | | 0.361 | | 0.165 | |
| Heart | 1.251 | 0.199 | 1.188 | | 0.185 | | 1.265 | | 0.163 | | 1.674 | | 0.165 | | 1.516 | | 0.168 | |
| Lung | 0.595 | 0.146 | 0.376 | | 0.165 | | 0.547 | | 0.192 | | 0.769 | | 0.127 | | 0.672 | | 0.166 | |
| Spleen | 7.778 | 0.141 | 8.574 | | 0.127 | | 7.592 | | 0.141 | | 6.600 | | 0.166 | | 6.815 | | 0.176 | |
| Pancreas | 0.652 | 0.197 | 0.603 | | 0.064 | | 0.744 | | 0.101 | | 0.798 | | 0.096 | | 0.689 | | 0.154 | |
| Liver | 8.903 | 0.125 | 12.100 | | 0.150 | | 6.545 | | 0.394 | | 11.954 | | 0.254 | | 9.907 | | 0.265 | |
| Stomach | 0.800 | 0.103 | 0.503 | | 0.157 | | 0.543 | | 0.106 | | 0.606 | | 0.116 | | 0.691 | | 0.122 | |
| Kidney | 1.319 | 0.412 | 1.243 | | 0.376 | | 1.176 | | 0.436 | | 1.313 | | 0.416 | | 1.275 | | 0.376 | |
| Bone | 0.651 | 0.026 | 1.018 | | 0.016 | | 0.859 | | 0.027 | | 1.651 | | 0.030 | | 1.367 | | 0.026 | |
| Small intestines | 1.021 | 0.149 | 1.156 | | 0.075 | | 1.237 | | 0.056 | | 1.103 | | 0.065 | | 0.950 | | 0.122 | |
| Tumour | 16.830 | 0.062 | 7.351 | | 0.073 | | 11.664 | | 0.056 | | 14.568 | | 0.065 | | 16.019 | | 0.035 | |
|  | **Treatment B (no pre-dosing)** | | | | | | | | | | | | | | | | | |
|  | Mouse 1 | | | Mouse 2 | | | | Mouse 3 | | | | Mouse 4 | | | | Mouse 5 | | |
|  | %IA/g | NWO (g) | | %IA/g | | NWO (g) | | %IA/g | | NWO (g) | | %IA/g | | NWO (g) | | %IA/g | | NWO (g) |
| Blood | 0.025 | 0.729 | | 0.032 | | 0.482 | | 0.045 | | 0.452 | | 0.034 | | 0.340 | | 0.078 | | 0.628 |
| Muscle | 0.273 | 0.175 | | 0.465 | | 0.039 | | 0.366 | | 0.148 | | 0.533 | | 0.058 | | 0.267 | | 0.117 |
| Heart | 1.257 | 0.161 | | 1.445 | | 0.197 | | 1.728 | | 0.178 | | 1.156 | | 0.181 | | 1.216 | | 0.185 |
| Lung | 0.359 | 0.241 | | 0.533 | | 0.198 | | 0.704 | | 0.151 | | 0.530 | | 0.091 | | 0.593 | | 0.052 |
| Spleen | 10.182 | 0.108 | | 6.889 | | 0.159 | | 10.239 | | 0.095 | | 7.603 | | 0.118 | | 7.574 | | 0.148 |
| Pancreas | 0.493 | 0.250 | | 0.370 | | 0.198 | | 0.493 | | 0.237 | | 0.411 | | 0.148 | | 0.764 | | 0.116 |
| Liver | 11.480 | 0.313 | | 9.474 | | 0.290 | | 8.109 | | 0.406 | | 8.649 | | 0.130 | | 11.126 | | 0.202 |
| Stomach | 0.443 | 0.126 | | 0.550 | | 0.131 | | 0.689 | | 0.115 | | 0.639 | | 0.107 | | 0.790 | | 0.127 |
| Kidney | 1.206 | 0.352 | | 1.270 | | 0.471 | | 1.040 | | 0.462 | | 1.099 | | 0.438 | | 1.308 | | 0.370 |
| Bone | 1.620 | 0.020 | | 1.946 | | 0.018 | | 1.805 | | 0.028 | | 0.917 | | 0.033 | | 1.395 | | 0.025 |
| Small intestines | 0.818 | 0.153 | | 0.721 | | 0.319 | | 1.036 | | 0.124 | | 0.912 | | 0.055 | | 1.490 | | 0.067 |
| Tumour | 10.653 | 0.040 | | 13.302 | | 0.028 | | 11.439 | | 0.064 | | 11.731 | | 0.079 | | 18.559 | | 0.008 |

Supplemental table 3. Ex vivo biodistribution data of mice that received ^89^Zr-DFO-FcRn(-)hG250 and ^225^Ac-DOTA-FcRn(-)hG250, reported are the percent injected activity per gram (%IA/g) and netto weight of the organs (NWO). (Data of Fig. 1).

|  | **^89^Zr-DFO-FcRn(-)hG250 4h** | | | | | | | | | |
| --- | --- | --- | --- | --- | --- | --- | --- | --- | --- | --- |
|  | Mouse 1 | | Mouse 2 | | Mouse 3 | | Mouse 4 | | Mouse 5 | |
|  | %IA/g | NWO (g) | %IA/g | NWO (g) | %IA/g | NWO (g) | %IA/g | NWO (g) | %IA/g | NWO (g) |
| Blood | 11.278 | 0.934 | 19.527 | 0.141 | 13.121 | 0.810 | 18.220 | 0.425 | 15.576 | 0.462 |
| Muscle | 0.969 | 0.085 | 1.809 | 0.060 | 0.703 | 0.074 | 0.671 | 0.131 | 0.740 | 0.145 |
| Heart | 5.991 | 0.140 | 7.234 | 0.110 | 5.686 | 0.108 | 9.918 | 0.131 | 6.220 | 0.127 |
| Lung | 10.526 | 0.133 | 11.551 | 0.153 | 13.260 | 0.139 | 12.123 | 0.139 | 10.174 | 0.152 |
| Spleen | 10.174 | 0.107 | 10.780 | 0.102 | 12.103 | 0.078 | 13.549 | 0.077 | 11.425 | 0.091 |
| Pancreas | 2.550 | 0.152 | 3.908 | 0.136 | 3.133 | 0.123 | 2.419 | 0.210 | 2.030 | 0.190 |
| Liver | 15.180 | 0.265 | 13.985 | 0.263 | 13.879 | 0.357 | 16.192 | 0.281 | 14.841 | 0.212 |
| Stomach | 2.003 | 0.102 | 2.065 | 0.132 | 2.179 | 0.125 | 2.481 | 0.100 | 2.390 | 0.095 |
| Kidney | 5.832 | 0.365 | 7.134 | 0.352 | 6.503 | 0.305 | 7.187 | 0.310 | 7.224 | 0.314 |
| Bone | 1.589 | 0.035 | 1.623 | 0.036 | 2.014 | 0.026 | 3.258 | 0.028 | 3.013 | 0.025 |
| Small intestines | 4.279 | 0.152 | 4.764 | 0.197 | 4.108 | 0.062 | 4.015 | 0.271 | 5.062 | 0.137 |
| Tumour | 10.386 | 0.036 | 8.926 | 0.079 | 11.060 | 0.028 | 9.025 | 0.090 | 7.339 | 0.073 |
|  | **^89^Zr-DFO-FcRn(-)hG250 24h** | | | | | | | | | |
|  | Mouse 1 | | Mouse 2 | | Mouse 3 | | Mouse 4 | | Mouse 5 | |
|  | %IA/g | NWO (g) | %IA/g | NWO (g) | %IA/g | NWO (g) | %IA/g | NWO (g) | %IA/g | NWO (g) |
| Blood | 3.536 | 0.368 | 4.766 | 0.329 | 5.115 | 0.221 | 3.843 | 0.282 | 5.234 | 0.209 |
| Muscle | 1.058 | 0.071 | 0.764 | 0.119 | 1.354 | 0.106 | 0.596 | 0.111 | 0.643 | 0.109 |
| Heart | 3.281 | 0.186 | 3.860 | 0.110 | 3.850 | 0.132 | 3.554 | 0.160 | 4.125 | 0.091 |
| Lung | 2.885 | 0.151 | 3.888 | 0.141 | 3.956 | 0.132 | 3.075 | 0.174 | 3.993 | 0.116 |
| Spleen | 14.993 | 0.077 | 17.759 | 0.071 | 19.927 | 0.062 | 14.662 | 0.098 | 20.103 | 0.055 |
| Pancreas | 1.739 | 0.096 | 1.477 | 0.155 | 2.119 | 0.075 | 1.045 | 0.133 | 1.554 | 0.142 |
| Liver | 14.001 | 0.328 | 17.394 | 0.237 | 18.826 | 0.285 | 20.292 | 0.230 | 15.106 | 0.734 |
| Stomach | 1.858 | 0.115 | 1.986 | 0.111 | 1.829 | 0.099 | 1.490 | 0.085 | 2.623 | 0.093 |
| Kidney | 2.696 | 0.358 | 3.354 | 0.342 | 3.318 | 0.311 | 3.067 | 0.318 | 3.513 | 0.295 |
| Bone | 1.779 | 0.041 | 2.222 | 0.018 | 2.933 | 0.019 | 2.417 | 0.018 | 1.931 | 0.015 |
| Small intestines | 2.966 | 0.124 | 3.414 | 0.094 | 2.403 | 0.096 | 2.414 | 0.163 | 5.553 | 0.024 |
| Tumour | 16.806 | 0.128 | 16.513 | 0.056 | 20.507 | 0.061 | 21.206 | 0.026 | 25.181 | 0.093 |
|  | **^89^Zr-DFO-FcRn(-)hG250 72h** | | | | | | | | | |
|  | Mouse 1 | | Mouse 2 | | Mouse 3 | | Mouse 4 | | Mouse 5 | |
|  | %IA/g | NWO (g) | %IA/g | NWO (g) | %IA/g | NWO (g) | %IA/g | NWO (g) | %IA/g | NWO (g) |
| Blood | 0.357 | 0.689 | 0.226 | 0.374 | 0.326 | 0.460 | 0.321 | 0.757 | 0.276 | 0.806 |
| Muscle | 0.461 | 0.118 | 0.310 | 0.122 | 0.433 | 0.090 | 1.023 | 0.124 | 0.435 | 0.147 |
| Heart | 2.799 | 0.112 | 2.123 | 0.111 | 2.640 | 0.117 | 2.166 | 0.121 | 2.569 | 0.108 |
| Lung | 1.480 | 0.150 | 0.825 | 0.088 | 1.442 | 0.129 | 1.297 | 0.120 | 1.363 | 0.149 |
| Spleen | 16.537 | 0.071 | 12.985 | 0.099 | 19.183 | 0.061 | 18.041 | 0.074 | 15.822 | 0.083 |
| Pancreas | 0.640 | 0.223 | 0.602 | 0.109 | 1.781 | 0.068 | 0.963 | 0.083 | 0.691 | 0.211 |
| Liver | 18.285 | 0.282 | 14.964 | 0.340 | 15.789 | 0.381 | 16.712 | 0.227 | 16.696 | 0.345 |
| Stomach | 1.306 | 0.078 | 0.803 | 0.126 | 1.364 | 0.130 | 1.170 | 0.091 | 1.195 | 0.079 |
| Kidney | 1.856 | 0.332 | 1.568 | 0.373 | 1.879 | 0.322 | 1.929 | 0.348 | 1.770 | 0.320 |
| Bone | 5.125 | 0.022 | 1.792 | 0.018 | 2.360 | 0.018 | 4.276 | 0.017 | 3.061 | 0.022 |
| Small intestines | 1.691 | 0.087 | 2.055 | 0.054 | 2.032 | 0.081 | 1.663 | 0.096 | 1.418 | 0.129 |
| Tumour | 17.529 | 0.108 | 17.578 | 0.174 | 30.107 | 0.025 | 28.765 | 0.023 | 16.015 | 0.073 |
|  | **^89^Zr-DFO-FcRn(-)hG250 168h** | | | | | | | | | |
|  | Mouse 1 | | Mouse 2 | | Mouse 3 | | Mouse 4 | | Mouse 5 | |
|  | %IA/g | NWO (g) | %IA/g | NWO (g) | %IA/g | NWO (g) | %IA/g | NWO (g) | %IA/g | NWO (g) |
| Blood | 0.077 | 0.397 | 0.079 | 0.483 | 0.080 | 0.489 | 0.068 | 0.373 | 0.061 | 0.527 |
| Muscle | 0.217 | 0.116 | 0.421 | 0.052 | 0.331 | 0.173 | 0.424 | 0.131 | 0.674 | 0.151 |
| Heart | 2.178 | 0.118 | 2.341 | 0.118 | 2.859 | 0.126 | 2.411 | 0.106 | 2.471 | 0.104 |
| Lung | 1.162 | 0.195 | 0.928 | 0.160 | 0.853 | 0.197 | 0.801 | 0.202 | 1.041 | 0.195 |
| Spleen | 13.243 | 0.100 | 23.295 | 0.065 | 17.204 | 0.089 | 13.512 | 0.093 | 20.502 | 0.069 |
| Pancreas | 1.178 | 0.131 | 1.535 | 0.098 | 0.558 | 0.164 | 0.547 | 0.125 | 0.671 | 0.164 |
| Liver | 18.066 | 0.542 | 20.941 | 0.287 | 16.625 | 0.214 | 20.230 | 0.198 | 17.714 | 0.237 |
| Stomach | 0.826 | 0.129 | 0.943 | 0.119 | 0.927 | 0.131 | 0.972 | 0.113 | 0.993 | 0.115 |
| Kidney | 1.357 | 0.377 | 1.481 | 0.357 | 1.539 | 0.341 | 1.420 | 0.314 | 1.425 | 0.321 |
| Bone | 2.500 | 0.013 | 2.559 | 0.026 | 2.692 | 0.035 | 2.577 | 0.038 | 3.003 | 0.035 |
| Small intestines | 1.147 | 0.132 | 2.167 | 0.103 | 1.999 | 0.121 | 0.842 | 0.246 | 1.641 | 0.136 |
| Tumour | 9.856 | 0.093 | 10.676 | 0.085 | 6.919 | 0.184 | 8.434 | 0.139 | 17.111 | 0.056 |
|  | **^225^Ac-DOTA-FcRn(-)hG250 4h** | | | | | | | | | |
|  | Mouse 1 | | Mouse 2 | | Mouse 3 | | Mouse 4 | | Mouse 5 | |
|  | %IA/g | NWO (g) | %IA/g | NWO (g) | %IA/g | NWO (g) | %IA/g | NWO (g) | %IA/g | NWO (g) |
| Blood | 15.847 | 0.735 | 19.137 | 0.209 | 18.519 | 0.214 | 20.904 | 0.208 | 18.079 | 0.292 |
| Muscle | 0.907 | 0.140 | 0.837 | 0.117 | 0.760 | 0.136 | 0.814 | 0.105 | 0.955 | 0.112 |
| Heart | 6.208 | 0.133 | 6.757 | 0.119 | 7.664 | 0.117 | 8.306 | 0.127 | 6.733 | 0.116 |
| Lung | 6.946 | 0.123 | 9.376 | 0.146 | 8.009 | 0.145 | 11.487 | 0.221 | 8.858 | 0.201 |
| Spleen | 7.635 | 0.113 | 8.715 | 0.115 | 8.376 | 0.090 | 7.641 | 0.094 | 7.477 | 0.109 |
| Pancreas | 1.405 | 0.181 | 1.494 | 0.178 | 2.111 | 0.128 | 2.152 | 0.149 | 1.909 | 0.133 |
| Liver | 20.006 | 0.280 | 21.711 | 0.264 | 22.352 | 0.205 | 21.321 | 0.223 | 20.463 | 0.228 |
| Stomach | 2.151 | 0.099 | 2.213 | 0.126 | 2.470 | 0.109 | 2.480 | 0.116 | 2.178 | 0.126 |
| Kidney | 10.551 | 0.341 | 6.018 | 0.312 | 5.975 | 0.306 | 6.936 | 0.332 | 5.363 | 0.323 |
| Bone | 5.136 | 0.020 | 4.321 | 0.040 | 5.569 | 0.028 | 3.598 | 0.050 | 3.762 | 0.059 |
| Small intestines | 2.775 | 0.225 | 3.684 | 0.252 | 3.085 | 0.210 | 3.787 | 0.140 | 3.940 | 0.113 |
| Tumour | 12.266 | 0.074 | 12.326 | 0.080 | 11.055 | 0.041 | 7.289 | 0.051 | 8.682 | 0.039 |
|  | **^225^Ac-DOTA-FcRn(-)hG250 24h** | | | | | | | | | |
|  | Mouse 1 | | Mouse 2 | | Mouse 3 | | Mouse 4 | | Mouse 5 | |
|  | %IA/g | NWO (g) | %IA/g | NWO (g) | %IA/g | NWO (g) | %IA/g | NWO (g) | %IA/g | NWO (g) |
| Blood | 3.312 | 0.209 | 3.734 | 0.405 | 2.833 | 0.375 | 2.574 | 0.210 | 2.523 | 0.326 |
| Muscle | 0.786 | 0.152 | 0.811 | 0.119 | 0.770 | 0.109 | 0.965 | 0.209 | 1.697 | 0.106 |
| Heart | 3.934 | 0.120 | 4.395 | 0.110 | 3.900 | 0.146 | 3.749 | 0.124 | 3.257 | 0.139 |
| Lung | 3.463 | 0.094 | 3.214 | 0.146 | 2.854 | 0.139 | 2.579 | 0.150 | 2.369 | 0.182 |
| Spleen | 12.134 | 0.080 | 15.904 | 0.069 | 11.475 | 0.067 | 12.704 | 0.091 | 11.937 | 0.072 |
| Pancreas | 1.485 | 0.182 | 1.199 | 0.148 | 1.104 | 0.145 | 0.916 | 0.192 | 0.918 | 0.138 |
| Liver | 23.885 | 0.240 | 22.917 | 0.166 | 23.811 | 0.331 | 19.784 | 0.218 | 23.686 | 0.239 |
| Stomach | 1.994 | 0.111 | 2.016 | 0.090 | 1.906 | 0.120 | 1.558 | 0.126 | 1.706 | 0.117 |
| Kidney | 2.610 | 0.326 | 2.680 | 0.300 | 2.724 | 0.285 | 2.386 | 0.341 | 2.275 | 0.320 |
| Bone | 5.123 | 0.019 | 6.997 | 0.022 | 5.689 | 0.054 | 3.533 | 0.045 | 5.080 | 0.046 |
| Small intestines | 2.046 | 0.172 | 1.806 | 0.189 | 1.714 | 0.142 | 2.250 | 0.140 | 1.924 | 0.160 |
| Tumour | 25.060 | 0.049 | 19.034 | 0.103 | 21.957 | 0.045 | 17.704 | 0.079 | 15.995 | 0.080 |
|  | **^225^Ac-DOTA-FcRn(-)hG250 72h** | | | | | | | | | |
|  | Mouse 1 | | Mouse 2 | | Mouse 3 | | Mouse 4 | | Mouse 5 | |
|  | %IA/g | NWO (g) | %IA/g | NWO (g) | %IA/g | NWO (g) | %IA/g | NWO (g) | %IA/g | NWO (g) |
| Blood | 0.338 | 0.127 | 0.230 | 0.288 | 0.120 | 0.485 | 0.143 | 0.373 | 0.204 | 0.504 |
| Muscle | 0.521 | 0.151 | 0.710 | 0.089 | 0.717 | 0.112 | 0.339 | 0.139 | 0.399 | 0.187 |
| Heart | 2.568 | 0.098 | 2.276 | 0.132 | 1.923 | 0.122 | 1.837 | 0.128 | 2.154 | 0.117 |
| Lung | 1.368 | 0.098 | 1.110 | 0.229 | 0.997 | 0.194 | 1.066 | 0.184 | 1.187 | 0.165 |
| Spleen | 20.928 | 0.040 | 20.407 | 0.034 | 15.889 | 0.052 | 18.510 | 0.037 | 19.096 | 0.052 |
| Pancreas | 0.854 | 0.085 | 0.632 | 0.157 | 0.630 | 0.145 | 0.607 | 0.189 | 0.539 | 0.173 |
| Liver | 26.539 | 0.268 | 21.391 | 0.332 | 20.066 | 0.238 | 18.232 | 0.306 | 14.281 | 0.335 |
| Stomach | 1.328 | 0.083 | 1.099 | 0.115 | 0.845 | 0.112 | 0.858 | 0.091 | 1.159 | 0.105 |
| Kidney | 1.483 | 0.292 | 1.399 | 0.335 | 1.412 | 0.344 | 1.203 | 0.331 | 1.377 | 0.323 |
| Bone | 5.906 | 0.016 | 4.745 | 0.038 | 4.640 | 0.039 | 6.687 | 0.019 | 4.789 | 0.043 |
| Small intestines | 1.287 | 0.110 | 1.336 | 0.128 | 1.050 | 0.126 | 1.119 | 0.163 | 1.299 | 0.160 |
| Tumour | 12.500 | 0.167 | 35.399 | 0.052 | 12.980 | 0.066 | 10.968 | 0.104 | 16.059 | 0.041 |
|  | **^225^Ac-DOTA-FcRn(-)hG250 168h** | | | | | | | | | |
|  | Mouse 1 | | Mouse 2 | | Mouse 3 | | Mouse 4 | | Mouse 5 | |
|  | %IA/g | NWO (g) | %IA/g | NWO (g) | %IA/g | NWO (g) | %IA/g | NWO (g) | %IA/g | NWO (g) |
| Blood | 0.048 | 0.414 | 0.084 | 0.476 | 0.072 | 0.265 | 0.055 | 0.570 | 0.065 | 0.448 |
| Muscle | 0.357 | 0.101 | 0.456 | 0.120 | 0.345 | 0.125 | 0.302 | 0.109 | 0.304 | 0.125 |
| Heart | 1.156 | 0.114 | 1.520 | 0.108 | 1.444 | 0.113 | 1.554 | 0.120 | 1.483 | 0.104 |
| Lung | 0.682 | 0.169 | 0.889 | 0.104 | 0.842 | 0.130 | 0.897 | 0.187 | 0.602 | 0.221 |
| Spleen | 27.229 | 0.030 | 31.713 | 0.029 | 25.800 | 0.033 | 32.656 | 0.020 | 30.850 | 0.025 |
| Pancreas | 0.426 | 0.191 | 0.422 | 0.165 | 0.387 | 0.178 | 0.424 | 0.156 | 0.632 | 0.179 |
| Liver | 15.675 | 0.290 | 17.485 | 0.256 | 15.174 | 0.362 | 18.359 | 0.316 | 17.696 | 0.227 |
| Stomach | 0.669 | 0.114 | 0.481 | 0.149 | 0.805 | 0.109 | 0.552 | 0.133 | 0.502 | 0.120 |
| Kidney | 0.768 | 0.329 | 0.973 | 0.321 | 0.876 | 0.342 | 0.758 | 0.304 | 0.747 | 0.308 |
| Bone | 4.281 | 0.016 | 3.306 | 0.036 | 3.573 | 0.059 | 4.832 | 0.053 | 5.674 | 0.019 |
| Small intestines | 0.586 | 0.144 | 0.869 | 0.263 | 0.814 | 0.112 | 0.917 | 0.113 | 0.580 | 0.137 |
| Tumour | 10.195 | 0.058 | 10.965 | 0.030 | 16.474 | 0.058 | 6.292 | 0.048 | 7.256 | 0.128 |

Supplemental table 4. Ex vivo biodistribution data of mice that received ^89^Zr-DFO-hG250 and ^225^Ac-DOTA-hG250, reported are the percent injected activity per gram (%IA/g) and netto weight of the organs (NWO). (Data of Fig. 2). The data on ^225^Ac-DOTA-hG250 is the data that was gathered as part of a previous publication by Merkx et al. [3]

|  | **^89^Zr-DFO-hG250 24h** | | | | | | | | | |
| --- | --- | --- | --- | --- | --- | --- | --- | --- | --- | --- |
|  | Mouse 1 | | Mouse 2 | | Mouse 3 | | Mouse 4 | | Mouse 5 | |
|  | %IA/g | NWO (g) | %IA/g | NWO (g) | %IA/g | NWO (g) | %IA/g | NWO (g) | %IA/g | NWO (g) |
| Blood | 20.056 | 0.218 | 19.139 | 0.466 | 18.262 | 0.195 | 14.757 | 0.378 | 15.037 | 0.419 |
| Muscle | 1.180 | 0.140 | 0.968 | 0.129 | 1.646 | 0.108 | 0.837 | 0.111 | 1.052 | 0.119 |
| Heart | 7.141 | 0.157 | 5.202 | 0.137 | 4.285 | 0.157 | 4.914 | 0.137 | 3.772 | 0.156 |
| Lung | 11.015 | 0.105 | 12.685 | 0.109 | 10.602 | 0.072 | 8.937 | 0.124 | 11.293 | 0.136 |
| Spleen | 4.654 | 0.081 | 5.484 | 0.074 | 5.082 | 0.079 | 3.549 | 0.139 | 3.983 | 0.120 |
| Pancreas | 3.391 | 0.107 | 2.597 | 0.165 | 2.640 | 0.211 | 2.348 | 0.166 | 2.460 | 0.202 |
| Liver | 5.141 | 0.293 | 5.735 | 0.288 | 6.116 | 0.211 | 4.346 | 0.332 | 4.993 | 0.244 |
| Stomach | 2.801 | 0.112 | 3.076 | 0.099 | 3.021 | 0.121 | 2.617 | 0.122 | 2.297 | 0.126 |
| Kidney | 6.908 | 0.361 | 7.084 | 0.371 | 6.696 | 0.389 | 5.204 | 0.382 | 5.492 | 0.365 |
| Bone | 3.603 | 0.045 | 4.221 | 0.045 | 3.238 | 0.047 | 3.114 | 0.037 | 3.443 | 0.041 |
| Small intestines | 3.668 | 0.193 | 3.689 | 0.231 | 3.076 | 0.156 | 4.017 | 0.091 | 3.101 | 0.178 |
| Tumour | 39.075 | 0.059 | 52.586 | 0.058 | 58.477 | 0.124 | 45.061 | 0.101 | 32.629 | 0.113 |
|  | **^89^Zr-DFO-hG250 72h** | | | | | | | | | |
|  | Mouse 1 | | Mouse 2 | | Mouse 3 | | Mouse 4 | | Mouse 5 | |
|  | %IA/g | NWO (g) | %IA/g | NWO (g) | %IA/g | NWO (g) | %IA/g | NWO (g) | %IA/g | NWO (g) |
| Blood | 10.934 | 0.102 | 12.808 | 0.125 | 8.871 | 0.281 | 13.427 | 0.430 | 11.252 | 0.096 |
| Muscle | 0.780 | 0.069 | 0.650 | 0.056 | 0.521 | 0.117 | 0.783 | 0.132 | 0.810 | 0.084 |
| Heart | 2.855 | 0.129 | 3.512 | 0.115 | 2.522 | 0.136 | 3.085 | 0.120 | 3.317 | 0.146 |
| Lung | 6.279 | 0.089 | 8.440 | 0.112 | 6.036 | 0.154 | 7.665 | 0.113 | 6.608 | 0.108 |
| Spleen | 3.743 | 0.128 | 5.272 | 0.078 | 4.685 | 0.104 | 3.971 | 0.108 | 2.885 | 0.141 |
| Pancreas | 2.436 | 0.112 | 2.488 | 0.094 | 2.082 | 0.108 | 2.495 | 0.128 | 1.944 | 0.153 |
| Liver | 3.927 | 0.396 | 9.324 | 0.283 | 5.513 | 0.490 | 5.648 | 0.610 | 4.306 | 0.468 |
| Stomach | 2.333 | 0.097 | 2.990 | 0.074 | 2.372 | 0.052 | 1.965 | 0.092 | 2.384 | 0.081 |
| Kidney | 4.826 | 0.411 | 6.072 | 0.250 | 4.140 | 0.383 | 5.877 | 0.360 | 5.021 | 0.431 |
| Bone | 5.207 | 0.030 | 8.111 | 0.011 | 8.201 | 0.012 | 7.175 | 0.031 | 5.171 | 0.032 |
| Small intestines | 4.033 | 0.076 | 4.991 | 0.034 | 3.197 | 0.076 | 4.019 | 0.062 | 2.614 | 0.133 |
| Tumour | 84.060 | 0.031 | 60.567 | 0.094 | 57.336 | 0.171 | 81.022 | 0.066 | 38.811 | 0.039 |
|  | **^89^Zr-DFO-hG250 168h** | | | | | | | | | |
|  | Mouse 1 | | Mouse 2 | | Mouse 3 | | Mouse 4 | | Mouse 5 | |
|  | %IA/g | NWO (g) | %IA/g | NWO (g) | %IA/g | NWO (g) | %IA/g | NWO (g) | %IA/g | NWO (g) |
| Blood | 5.643 | 0.509 | 5.420 | 0.363 | 6.324 | 0.068 | 4.424 | 0.225 | 4.923 | 0.214 |
| Muscle | 0.464 | 0.097 | 0.530 | 0.108 | 0.545 | 0.048 | 0.401 | 0.136 | 0.456 | 0.099 |
| Heart | 2.026 | 0.149 | 1.550 | 0.124 | 1.672 | 0.140 | 1.531 | 0.158 | 1.859 | 0.117 |
| Lung | 4.261 | 0.147 | 3.515 | 0.056 | 3.950 | 0.116 | 3.509 | 0.118 | 3.792 | 0.077 |
| Spleen | 3.348 | 0.145 | 3.791 | 0.107 | 3.778 | 0.121 | 3.080 | 0.137 | 4.925 | 0.112 |
| Pancreas | 1.210 | 0.101 | 1.062 | 0.273 | 1.167 | 0.151 | 0.892 | 0.288 | 0.840 | 0.237 |
| Liver | 5.480 | 0.378 | 5.900 | 0.231 | 4.617 | 0.185 | 5.008 | 0.211 | 4.926 | 0.135 |
| Stomach | 1.262 | 0.129 | 1.261 | 0.100 | 1.570 | 0.091 | 1.078 | 0.126 | 1.300 | 0.088 |
| Kidney | 3.128 | 0.411 | 3.589 | 0.406 | 3.346 | 0.425 | 2.834 | 0.449 | 3.393 | 0.332 |
| Bone | 10.137 | 0.038 | 10.326 | 0.042 | 8.402 | 0.026 | 9.046 | 0.034 | 12.773 | 0.030 |
| Small intestines | 1.990 | 0.051 | 1.214 | 0.166 | 1.536 | 0.214 | 1.069 | 0.255 | 1.653 | 0.106 |
| Tumour | 89.467 | 0.078 | 62.210 | 0.128 | 86.095 | 0.051 | 73.679 | 0.090 | 83.563 | 0.087 |
|  | **^225^Ac-DOTA-hG250 24h** | | | | | | | | | |
|  | Mouse 1 | | Mouse 2 | | Mouse 3 | | Mouse 4 | | Mouse 5 | |
|  | %IA/g | NWO (g) | %IA/g | NWO (g) | %IA/g | NWO (g) | %IA/g | NWO (g) | %IA/g | NWO (g) |
| Blood | 17.713 | 0.330 | 18.287 | 0.385 | 19.244 | 0.153 | 19.532 | 0.052 | 18.746 | 0.112 |
| Muscle | 1.418 | 0.090 | 0.864 | 0.100 | 0.948 | 0.127 | 1.182 | 0.122 | 1.466 | 0.129 |
| Heart | 5.735 | 0.137 | 5.604 | 0.131 | 6.029 | 0.132 | 4.957 | 0.187 | 7.664 | 0.159 |
| Lung | 9.299 | 0.088 | 8.852 | 0.155 | 13.095 | 0.202 | 13.520 | 0.197 | 12.311 | 0.257 |
| Spleen | 5.450 | 0.072 | 6.083 | 0.073 | 6.185 | 0.078 | 5.928 | 0.070 | 5.228 | 0.064 |
| Pancreas | 2.258 | 0.188 | 2.617 | 0.193 | 3.586 | 0.256 | 2.602 | 0.189 | 1.686 | 0.128 |
| Liver | 8.894 | 0.407 | 11.075 | 0.410 | 9.884 | 0.502 | 9.113 | 0.256 | 11.733 | 0.415 |
| Stomach | 2.404 | 0.106 | 2.317 | 0.108 | 3.001 | 0.109 | 2.791 | 0.065 | 2.624 | 0.125 |
| Kidney | 5.779 | 0.358 | 5.893 | 0.341 | 5.994 | 0.342 | 6.274 | 0.342 | 6.182 | 0.332 |
| Bone | 3.835 | 0.055 | 4.024 | 0.049 | 2.788 | 0.047 | 3.384 | 0.069 | 3.467 | 0.051 |
| Small intestines | 2.624 | 0.149 | 2.934 | 0.195 | 2.924 | 0.327 | 3.589 | 0.165 | 3.474 | 0.145 |
| Tumour | 72.798 | 0.052 | 36.843 | 0.038 | 35.545 | 0.083 | 32.277 | 0.062 | 43.224 | 0.158 |
|  | **^225^Ac-DOTA-hG250 72h** | | | | | | | | | |
|  | Mouse 1 | | Mouse 2 | | Mouse 3 | | Mouse 4 | | Mouse 5 | |
|  | %IA/g | NWO (g) | %IA/g | NWO (g) | %IA/g | NWO (g) | %IA/g | NWO (g) | %IA/g | NWO (g) |
| Blood | 16.396 | 0.378 | 15.139 | 0.173 | 10.963 | 0.250 | 13.639 | 0.082 | 13.122 | 0.192 |
| Muscle | 0.933 | 0.082 | 0.828 | 0.080 | 1.596 | 0.075 | 0.982 | 0.121 | 1.069 | 0.064 |
| Heart | 6.055 | 0.128 | 5.470 | 0.145 | 3.648 | 0.132 | 3.295 | 0.119 | 4.079 | 0.133 |
| Lung | 7.377 | 0.183 | 7.903 | 0.171 | 6.100 | 0.078 | 7.049 | 0.087 | 8.759 | 0.105 |
| Spleen | 8.864 | 0.027 | 10.024 | 0.031 | 6.415 | 0.048 | 10.198 | 0.041 | 9.524 | 0.057 |
| Pancreas | 1.665 | 0.134 | 2.024 | 0.147 | 2.459 | 0.139 | 3.338 | 0.102 | 2.622 | 0.247 |
| Liver | 10.520 | 0.253 | 8.537 | 0.240 | 8.695 | 0.241 | 9.808 | 0.161 | 6.652 | 0.349 |
| Stomach | 2.202 | 0.122 | 2.833 | 0.124 | 2.068 | 0.122 | 2.806 | 0.113 | 3.569 | 0.115 |
| Kidney | 4.350 | 0.320 | 3.844 | 0.340 | 3.252 | 0.366 | 3.638 | 0.315 | 3.760 | 0.367 |
| Bone | 3.746 | 0.071 | 3.868 | 0.064 | 4.241 | 0.048 | 5.366 | 0.049 | 6.722 | 0.065 |
| Small intestines | 3.636 | 0.132 | 3.551 | 0.110 | 1.949 | 0.314 | 3.188 | 0.220 | 3.442 | 0.189 |
| Tumour | 111.821 | 0.032 | 156.572 | 0.062 | 115.943 | 0.084 | 109.353 | 0.118 | 59.137 | 0.027 |
|  | **^225^Ac-DOTA-hG250 168h** | | | | | | | | | |
|  | Mouse 1 | | Mouse 2 | | Mouse 3 | | Mouse 4 | | Mouse 5 | |
|  | %IA/g | NWO (g) | %IA/g | NWO (g) | %IA/g | NWO (g) | %IA/g | NWO (g) | %IA/g | NWO (g) |
| Blood | 0.722 | 0.152 | 5.512 | 0.120 | 7.686 | 0.082 | 3.596 | 0.370 | 5.978 | 0.325 |
| Muscle | 0.099 | 0.050 | 0.366 | 0.070 | 0.421 | 0.078 | 0.319 | 0.094 | 0.489 | 0.129 |
| Heart | 0.466 | 0.130 | 2.087 | 0.116 | 3.789 | 0.152 | 1.702 | 0.145 | 4.518 | 0.124 |
| Lung | 0.988 | 0.218 | 4.801 | 0.155 | 6.801 | 0.236 | 3.994 | 0.127 | 2.654 | 0.103 |
| Spleen | 13.687 | 0.014 | 17.679 | 0.018 | 18.218 | 0.015 | 0.913 | 0.170 | 14.273 | 0.018 |
| Pancreas | 0.233 | 0.080 | 1.390 | 0.087 | 1.184 | 0.138 | 5.505 | 0.047 | 1.250 | 0.188 |
| Liver | 13.077 | 0.215 | 10.835 | 0.213 | 8.134 | 0.249 | 8.831 | 0.250 | 10.563 | 0.164 |
| Stomach | 0.598 | 0.104 | 2.036 | 0.095 | 1.670 | 0.100 | 1.379 | 0.079 | 1.625 | 0.074 |
| Kidney | 1.217 | 0.270 | 2.178 | 0.295 | 2.475 | 0.330 | 1.732 | 0.323 | 2.139 | 0.268 |
| Bone | 2.476 | 0.067 | 5.323 | 0.048 | 5.090 | 0.048 | 4.406 | 0.051 | 4.222 | 0.053 |
| Small intestines | 0.285 | 0.241 | 1.440 | 0.114 | 1.772 | 0.315 | 0.901 | 0.472 | 1.416 | 0.236 |
| Tumour | 140.3 | 0.191 | 264.4 | 0.054 | 255.0 | 0.046 | 193.0 | 0.053 | 211.5 | 0.065 |

Supplemental table 5. Dosimetry of ^225^Ac-FcRn(-)hG250. measured and predicted based on ^89^Zr-FcRn(-)hG250 in xenografted mice using OLINDA/EXM 2.1. MAD = Measured Absorbed Dose. PAD = Predicted Absorbed Dose. RMSE = Root Mean Square Error. Gy = Gray. Bq = Bequerel. Deviation is difference in Gy between measured and predicted absorbed dose. with the percentage deviation of measured dose between brackets. *Organs that are included in OLINDA/EXM but were no part of the biodistribution are excluded. (Data of Fig. 3).

| Organ* | MAD ± RMSE  (mGy/kBq) | PAD **±** RMSE  (mGy/kBq) | MAD (25 kBq) ± RMSE  (Gy) | PAD (25 kBq) ± RMSE  (Gy) | Deviation ± SD (Gy) | Deviation (%) |
| --- | --- | --- | --- | --- | --- | --- |
| Small Intestines | 389 ± 94 | 550 ± 307 | 9.7 ± 2.4 | 13.8 ± 7.7 | 4.0 ± 2.4 | 41.4 |
| Stomach | 305 ± 105 | 348 ± 154 | 7.6 ± 2.6 | 8.7 ± 3.9 | 1.1 ± 0.6 | 14.1 |
| Heart | 653 ± 223 | 805 ± 325 | 16.3 ± 5.6 | 20.1 ± 8.1 | 3.8 ± 2.0 | 23.3 |
| Kidneys | 474 ± 208 | 579 ± 336 | 11.9 ± 5.2 | 14.5 ± 8.4 | 2.6 ± 1.9 | 22.2 |
| Liver | 4225 ± 1618 | 5070 ± 1547 | 105.6 ± 40.5 | 126.8 ± 38.7 | 21.1 ± 10.3 | 20.0 |
| Lungs | 468 ± 188 | 508 ± 183 | 11.7 ± 4.7 | 12.7 ± 4.6 | 1.0 ± 0.5 | 8.5 |
| Pancreas | 258 ± 110 | 340 ± 228 | 6.5 ± 2.8 | 8.5 ± 5.7 | 2.1 ± 1.6 | 31.8 |
| Skeleton | 98 ± 43 | 60 ± 28 | 2.5 ± 1.1 | 1.5 ± 0.7 | -1.0 ± -0.6 | -38.8 |
| Spleen | 4084 ± 2088 | 2727 ± 1434 | 102.1 ± 52.2 | 68.2 ± 35.9 | -33.9 ± -24.9 | -33.2 |
| Total Body | 547 ± 315 | 547 ± 343 | 13.7 ± 7.9 | 13.7 ± 8.6 | 0.0 ± 0.0 | 0.0 |
| Tumour | 2496 ± 972 | 2651 ± 857 | 62.4 ± 24.3 | 66.3 ± 21.4 | 3.9 ± 2.0 | 6.2 |

Supplemental table 6. Dosimetry of ^225^Ac-hG250. measured and predicted based on ^89^Zr- hG250 in xenografted mice using OLINDA/EXM 2.1. MAD = Measured Absorbed Dose. PAD = Predicted Absorbed Dose. RMSE = Root Mean Square Error. Gy = Gray. Bq = Bequerel. Deviation is difference in Gy between measured and predicted absorbed dose. with the percentage deviation of measured dose between brackets. *Organs that are included in OLINDA/EXM but were no part of the biodistribution are excluded. (Data of Fig. 3).

| Organ* | MAD ± RMSE  (mGy/kBq) | PAD **±** RMSE  (mGy/kBq) | MAD (50 kBq) ± RMSE  (Gy) | PAD (50 kBq) ± RMSE  (Gy) | Deviation ± SD (Gy) | Deviation (%) |
| --- | --- | --- | --- | --- | --- | --- |
| Small Intestines | 453 ± 228 | 353 ± 157 | 22.7 ± 11.4 | 17.7 ± 7.9 | -5.0 ± -3.4 | -22.1 |
| Stomach | 291 ± 89 | 390 ± 130 | 14.6 ± 4.5 | 19.5 ± 6.5 | 5.0 ± 2.2 | 34.0 |
| Heart | 721 ± 155 | 503 ± 172 | 36.1 ± 7.8 | 25.1 ± 8.6 | -10.9 ± -4.4 | -30.2 |
| Kidneys | 612 ± 73 | 834 ± 318 | 30.6 ± 3.7 | 41.7 ± 15.9 | 11.1 ± 4.4 | 36.3 |
| Liver | 2338 ± 466 | 1099 ± 371 | 116.9 ± 23.3 | 55.0 ± 18.6 | -62.0 ± -24.3 | -53.0 |
| Lungs | 1118 ± 200 | 1047 ± 359 | 55.9 ± 10.0 | 52.4 ± 18.0 | -3.6 ± -1.4 | -6.4 |
| Pancreas | 384 ± 312 | 353 ± 135 | 19.2 ± 15.6 | 17.6 ± 6.8 | -1.6 ± -1.4 | -8.1 |
| Skeleton | 91 ± 21 | 77 ± 25 | 4.6 ± 1.1 | 3.8 ± 1.3 | -0.7 ± -0.3 | -15.4 |
| Spleen | 1333 ± 629 | 856 ± 265 | 66.7 ± 31.5 | 42.8 ± 13.3 | -23.9 ± -13.5 | -35.8 |
| Total Body | 375 ± 86 | 386 ± 158 | 18.7 ± 4.3 | 19.3 ± 7.9 | 0.6 ± 0.3 | 2.9 |
| Tumour | 40883 ± 10234 | 12609 ± 4639 | 2044.2 ± 511.7 | 630.4 ± 232.0 | -1413.7 ± -629.1 | -69.2 |

Supplemental table 7. Baseline animal characteristics

| **hG250 study** | | | | | | | | | | | | | |
| --- | --- | --- | --- | --- | --- | --- | --- | --- | --- | --- | --- | --- | --- |
| **Characteristic** | **^225^Ac**  **24h** | | **^225^Ac**  **72h** | | **^225^Ac**  **168h** | | | **^89^Zr**  **24h** | | **^89^Zr**  **72h** | | **^89^Zr**  **168h** | |
| BALB/cAnN-foxn1nu/Rj // (n) | 5 | | 5 | | 5 | | | 5 | | 5 | | 5 | |
| Age at treatment (weeks) | 9.7 | | 9.7 | | 9.7 | | | 9.7 | | 9.7 | | 9.7 | |
| Weight at treatment (g) | 20.5 ± 0.4 | | 21.2 ± 1.9 | | 20.8 ± 0.3 | | | 21.8 ± 1.0 | | 21.1 ± 1.4 | | 21.9 ± 1.7 | |
| Tumor volume treatment (mm^3^) | 72.8 ± 33.3 | | 64.7 ± 34.8 | | 72.5 ± 28.6 | | | 69.9 ± 33.0 | | 71.9 ± 35.6 | | 65.8 ± 33.6 | |
|  |  | |  | |  | | |  | |  | |  | |
| **FcRn(-)hG250 study** | | | | | | | | | | | | | |
| **Characteristic** | **^225^Ac**  **4h** | **^225^Ac**  **24h** | | **^225^Ac**  **72h** | | **^225^Ac**  **168h** | **^89^Zr**  **4h** | | **^89^Zr**  **24h** | | **^89^Zr**  **72h** | | **^89^Zr**  **168h** |
| BALB/cAnN-foxn1nu/Rj // (n) | **5** | 5 | | 5 | | 5 | 5 | | 5 | | 5 | | 5 |
| Age at treatment (weeks) | 11.7 | 11.7 | | 11.7 | | 11.7 | 11.7 | | 11.7 | | 11.7 | | 11.7 |
| Weight at treatment (g) | 20.8 ± 0.5 | 20.9 ± 1.0 | | 20.4 ± 1.2 | | 20.9 ± 0.9 | 21.4 ± 0.5 | | 21.1 ± 1.3 | | 19.9 ± 0.7 | | 21.5 ± 1.3 |
| Tumor volume treatment (mm^3^) | 83.5 ± 35.6 | 85.0 ± 40.2 | | 87.7 ± 39.0 | | 83.1 ± 34.9 | 86.0 ± 35.1 | | 76.6 ± 33.3 | | 90.0 ± 42.3 | | 73.7 ± 32.8 |

Continuous data are mean and standard deviation

**References**

1.	Merkx RIJ, Lobeek D, Konijnenberg M, Jiménez-Franco LD, Kluge A, Oosterwijk E, et al. Phase I study to assess safety, biodistribution and radiation dosimetry for (89)Zr-girentuximab in patients with renal cell carcinoma. Eur J Nucl Med Mol Imaging. 2021;48:3277-85. doi:10.1007/s00259-021-05271-w.

2.	Lewis MR, Raubitschek A, Shively JE. A facile, water-soluble method for modification of proteins with DOTA. Use of elevated temperature and optimized pH to achieve high specific activity and high chelate stability in radiolabeled immunoconjugates. Bioconjug Chem. 1994;5:565-76. doi:10.1021/bc00030a012.

3.	Merkx RIJ, Rijpkema M, Franssen GM, Kip A, Smeets B, Morgenstern A, et al. Carbonic Anhydrase IX-Targeted α-Radionuclide Therapy with 225Ac Inhibits Tumor Growth in a Renal Cell Carcinoma Model. Pharmaceuticals (Basel). 2022;15. doi:10.3390/ph15050570.
